# Random Whole Metagenomic Sequencing for Forensic Discrimination of Soils

**DOI:** 10.1371/journal.pone.0104996

**Published:** 2014-08-11

**Authors:** Anastasia S. Khodakova, Renee J. Smith, Leigh Burgoyne, Damien Abarno, Adrian Linacre

**Affiliations:** 1 School of Biological Sciences, Flinders University, Adelaide, Australia; 2 Forensic Science South Australia, Adelaide, Australia; Institut de Biologia Evolutiva - Universitat Pompeu Fabra, Spain

## Abstract

Here we assess the ability of random whole metagenomic sequencing approaches to discriminate between similar soils from two geographically distinct urban sites for application in forensic science. Repeat samples from two parklands in residential areas separated by approximately 3 km were collected and the DNA was extracted. Shotgun, whole genome amplification (WGA) and single arbitrarily primed DNA amplification (AP-PCR) based sequencing techniques were then used to generate soil metagenomic profiles. Full and subsampled metagenomic datasets were then annotated against M5NR/M5RNA (taxonomic classification) and SEED Subsystems (metabolic classification) databases. Further comparative analyses were performed using a number of statistical tools including: hierarchical agglomerative clustering (CLUSTER); similarity profile analysis (SIMPROF); non-metric multidimensional scaling (NMDS); and canonical analysis of principal coordinates (CAP) at all major levels of taxonomic and metabolic classification. Our data showed that shotgun and WGA-based approaches generated highly similar metagenomic profiles for the soil samples such that the soil samples could not be distinguished accurately. An AP-PCR based approach was shown to be successful at obtaining reproducible site-specific metagenomic DNA profiles, which in turn were employed for successful discrimination of visually similar soil samples collected from two different locations.

## Introduction

Soil can be found on items submitted for forensic analysis, however there is currently no reliable method to compare the DNA content of soils for forensic purposes. Soil, owing to its inherent features, adheres under fingernails, to cars, tools, weapons or items of clothing and can transfer during the commission of a criminal act [1]. Soil can also be useful associative evidence in the investigation of wildlife crimes, such as poaching. The presence of soil is often recorded during the forensic examination of exhibits. Due to the lack of a validated analytical method, or set of techniques for meaningful comparison of soil samples, this evidential type provides only limited value in investigations. There is therefore a need to develop such comparative methodologies.

Traditionally forensic analysis of soils involves comparison of its chemical-physical and biological properties [2]. Over the past decades many studies have been undertaken utilizing the chemical profiles of soil using a wide variety of novel sophisticated and rapid analytical methods such as, FTIR [3], X-ray [4] and elemental analysis [5], [6]. These methods are mainly mineralogical techniques and define geological characteristics of soil, which differ across a regional scale. Therefore these techniques may be unable to discriminate soils within a small locality [7]. The potential for discriminating soils at a local scale exists with methods of soil microbial community analysis that have been applied for forensic purposes [8], [9]. Previous attempts at DNA based analysis of soils used DNA fingerprinting techniques which evaluate fragment length variation such as terminal restriction fragment length polymorphism (TRFLP) [7], [10], denaturing gradient gel electrophoresis (DGGE) [11], amplified ribosomal DNA restriction analysis (ARDRA) [12] and length heterogeneity-polymerase chain reaction (LH-PCR) [5]. Many fragments in the resultant DNA fingerprint appear identical in length but differ in sequence leading to erroneous conclusions of similarity that would be avoided if the DNA sequences of the fragments were known. These methods are rapid and permit high throughput analysis but have insufficient resolution to discriminate complex soil mixtures [13]. All these methods have potential for use in forensic comparisons, however a lack of reproducibility and the potential for false inclusions has restricted their implementation in a forensic setting.

Development of new platforms for high-throughput DNA sequencing (HTS) has made it more affordable and led to the significant growth of HTS-based studies [14]–[16]. The application of HTS to soil science has allowed for new insight on the diversity of soil microbial communities inhabiting various biomes [17]–[19].

Gene-targeted, or locus-specific, sequencing which typically targets the 16S rRNA gene is used for characterization of the taxonomic composition and diversity of microbial communities [20], [21]. Shotgun sequencing is primarily a method for studying the functional structure of the communities which aims to examine the entire genetic assemblage and, being amplification-independent, relies on variation and commonality of the collective genomes found in a given environmental sample [22], [23]. Shotgun typing allows for a more comprehensive perspective on the whole microbial community but is limited by its propensity to favour identification of the most dominant members over rarer organisms [24]. In order to access the rare species found in such a complex matrix as soil, ultra-deep DNA sequencing is required [25].

Soil samples obtained during forensic investigations, by their nature, put specific requirements on any metagenomic approach. The samples are often small and sufficient amount of the sample should remain after analysis for independent re-testing if required. Soil DNA extraction procedure, as an initial step of metagenomic analysis, should provide high quality DNA with a good yield. Commercially available soil DNA extraction kits is an preferable option offering forensic investigators a means for standardizing soil DNA extraction [26]–[28]. Gene-targeted sequencing based on PCR amplification technique is able to analyse the minute amounts of template DNA recovered in forensic samples. The need for a relatively large amount of initial DNA template for shotgun sequencing makes this approach less suitable for forensic oriented metagenomic analysis but whole-genome amplification (WGA), using Phi 29 DNA polymerase, represents an effective way of enabling whole-genome shotgun sequencing from small quantities of DNA [29].

The ability to identify DNA from the entire genetic composition of a complex soil mixture is desirable for forensic investigation as the DNA from a wide range of organisms may be present: these include the DNA from bacteria, fungi, nematodes, mammals, plant material, and from insect remains. These can be used to generate a rich DNA profile for comparison and meaningful discrimination between samples. Targeted metagenomics technique that are limited to one particular locus, such as the 16S (small subunit (SSU) of rRNA in prokaryotes [30]), ITS (internal transcribed spacers widely used for fungi [31]), or 18S (nuclear SSU rRNA a widely used phylogenetic marker in eukaryotes [32]), do not detect the variability of the entire soil biota thus providing less information for comparison and differentiation of soil samples.

Metagenomic sequencing approaches have been reported that can reliably differentiate soil microbial communities from different soil types and different land use [17], [33]. However from a forensic point of view, discrimination of visually similar soil samples taken from geographically different urban areas (community parks with similar plant cover, residential suburbs, soils of similar land management) is of greater importance [7], [34].

We report on the assessment of the ability of random whole metagenomic sequencing approaches to produce reproducible site-specific DNA profiles that can be employed for comparative analysis and discrimination between soils of different locality for application in forensic science. We show an assessment of shotgun and WGA-based sequencing techniques as well as the use of a single arbitrarily primed DNA amplification (AP-PCR) for metagenomic soil DNA analysis [35]. The use of AP-PCR was first reported in the 1990s [36], [37] and has been applied to genotyping [38], [39] and the study of microbial communities [40].

## Materials and Methods

### Soil sampling

Soil samples were collected from two different sites in Adelaide in July 2013; Location A (S35 01 43.42 E138 34 16.26) and Location B (S35 00 58.09 E138 32 12.03). These locations are separated by approximately 3 km. For each site, triplicate samples were taken 1 m apart from the upper 1 cm of the soil layer. The samples collected from Location A and Location B represented a dark loam rich in organic matter and were visually very similar. No specific permits were required for these locations and activities. The field studies did not involve endangered or protected species. The soil samples were placed in individual sterile plastic tubes and stored at −20°C until analysis. DNA extraction was performed within 24 hours of soil sampling.

### DNA extraction, amplification and sequencing

Metagenomic DNA was isolated from 50 mg of each soil sample using the ZR Soil Microbe DNA Kit (Zymo Research, USA) following the manufacturer’s recommendations. The quality of the DNA extracts was verified by gel electrophoresis in a 1% agarose gel stained with ethidium bromide. DNA concentrations were determined using a Qubit dsDNA HS Assay Kit (Invitrogen, USA) on a Qubit fluorometer (Life technologies, USA).

For each of the six samples, WGA was conducted with 20 ng DNA using Phi29 DNA polymerase (REPLI-g, Qiagen, Germany). The quality of amplification products was determined by 1% agarose gel electrophoresis and by quantification on a Qubit fluorometer (Life technologies, USA) after purification with a QIAquick PCR Kit (Qiagen, Germany).

Amplification of extracted soil DNA was performed with an arbitrarily chosen oligonucleotide primer (sequence 5′-GGAGGTGGTGTTCGAGGG-3′), previously reported for generating soil DNA fingerprints [41]. As a template, 4 ng of metagenomic DNA was used. The 25 µL final reaction volume contained 1×Hotstar Taq buffer (Qiagen, Germany), 2.5 mM Mg^2+^, 0.2 mM of each dNTPs, 0.4 µM of the arbitrary chosen primer, and 0.5 U HotstarTaq DNA polymerase (Qiagen, Germany). An initial 15 min denaturation step at 95 °C was followed by 42 cycles of 30 s at 94°C, 30 s at 55°C and 1 min at 72°C. A final extension step of 7 min at 72°C was used to complete the reaction. The quality and concentration of purified PCR products (QIAquick PCR Kit, Qiagen, Germany) were determined as described for WGA procedure.

All the manipulations were performed in dedicated DNA extraction and PCR-mixing hoods using sterile DNA/RNA free water (Ambion, USA) and DNA/RNA free plasticware (Eppendorf, Germany). All the procedures of the extraction and amplification were conducted with the necessary no-template controls, including extraction blank controls.

Library preparation from 100 ng of the corresponding DNA specimen for all three methods under evaluation followed by sequencing was performed at the Australian Genome Research Facility (AGRF, http://www.agrf.org.au/, Adelaide, SA, Australia) using Ion Torrent technology (Ion Torrent PGM Sequencer; Life Technologies, USA) on a separate Ion 318 chip for each of the sequencing approaches.

### Processing of sequencing data

Raw sequence datasets were uploaded to the Metagenome Rapid Annotation using Subsystem Technology (MG-RAST) server (http://metagenomics.nmpdr.org/) (Meyer et al., 2008) and filtered from low-quality reads prior to annotation. Metagenomic datasets were annotated to protein genes against the M5NR database and SEED Subsystems database resulting in protein-derived taxonomic and metabolic profiles, respectively. In addition taxonomic profiles were generated by comparison of the metagenomic datasets with the M5RNA ribosomal database also available in MG-RAST. The MG-RAST default annotation parameters such as maximum E-value <1×10^−5^, minimum length of alignment of 15 bp, and minimum sequence identity of 60%, were used to identify the best database matches. Metagenomic profiles were generated at all available MG-RAST taxonomic (phylum to species) and metabolic (level 1 to functions) levels of hierarchy. To adjust the differences in sequencing effort across samples, two common procedure of standardization were taken:

1. In the first approach metagenomic profiles were generated using full datasets of the high-quality reads obtained for each sample. For the metagenomic profiles comparison the relative abundance scores for each taxon and metabolic feature were determined by the percentages of respective reads over the total assigned reads. In the text the relative abundance scores found both for the taxonomic and metabolic features are represented as an average ± SD (standard deviation) across all datasets (if not mentioned otherwise).
2. A second approach was based on comparison of metagenomic profiles generated from randomly subsampled datasets of 49 000 annotated reads per sample.

Metagenomic datasets are freely available on the MG-RAST web-server (http://metagenomics.anl.gov/). The MG-RAST sample IDs are listed in the Table S1.

### Statistical analysis of data

The species richness was estimated by rarefaction analysis preformed in MG-RAST. The analysis was performed for total taxa identified with the M5NR protein database in randomly subsampled metagenomic datasets (including Bacteria, Archaea, Eukaryota, Viruses, unclassified and other sequences).

Statistical comparison of metagenomes was conducted on square root transformed data using the statistical package Primer v.6 for Windows (Version 6.1.13, PRIMER-E, Plymouth) [43]. To assess the similarity of the taxonomic and metabolic compositions between soil samples, the Bray-Curtis pair-wise similarity measure was employed. The resulting Bray-Curtis similarity matrices were then used for hierarchical agglomerative clustering (CLUSTER) with the results displayed as group average dendrograms. Similarity profile analysis (SIMPROF) was used to test for multivariate structure in the clusters formed. Non-metric multi-dimensional scaling (NMDS) of Bray-Curtis similarities was performed as an unconstrained ordination method to graphically visualise inter-sample relationships. The program RELATE in the PRIMER package was used to calculate the Spearman rank correlation between Bray-Curtis similarity matrices generated from differently standardised datasets at the same level of taxonomic or metabolic resolution [44].

Metagenome profiles were further analysed using canonical analysis of principal coordinates (CAP) using the PERMANOVA+ version 1.0.3 3 add-on to PRIMER [45] as a constrained ordination method to test for significant differences among the *a priori* groups in multivariate space. All metagenomic profiles were divided into 6 groups according to the sequencing approach applied and origin of the samples. The *a priori* hypothesis of ‘no difference’ within groups was tested at both taxonomic and metabolic levels using CAP analysis by evaluation of a *P*-value obtained after 9999 permutations. The strength of the association between multivariate data and the hypothesis of group differences was indicated by the value of the squared canonical correlation (δ_1_^2^). An appropriate number of principal coordinates axes (*m*) used for the CAP analysis were chosen automatically by the CAP routine to minimize errors of a misclassification. In order to validate the ability of the CAP model to classify correctly the samples according to their appropriate groups a cross-validation procedure was performed for the chosen value of *m* [46].

## Results

### Notation and general characteristics of sequencing datasets

Obtained datasets were grouped and named according to their sequencing approach and soil sampling sites. Thus samples processed by the AP-PCR approach have a common prefix “AP”, shotgun sequenced samples – “SH”, and WGA assisted sequencing – “WGA”. Each dataset designation identifies the location from where the sample was collected: “_A” – samples collected from location A; and “_B” – samples collected from location B. For example the abbreviation AP_A indicates a sample collected at location A and sequenced by the AP-PCR-based method.

For each soil DNA sample three datasets were generated from the same DNA template using three sequencing approaches. Shotgun metagenome sequencing resulted in an average of 672 542 (531 108–806 843) sequence reads with an average sequence length of 198±73 bases for a total of >133 Mbp of sequence. Sequencing datasets after WGA consisted of an average of 911 554 (506 028–2 012 359) sequences with an average of 198±75 bases in length for a total of >178 Mbp. The AP-based approach gave an average of 468 187 (74 370–1 047 266) reads with an average 143±69 bases in length for a total of >70.7 Mbp (Table 1). Datasets were annotated using the online MG-RAST server [42]. Approximately 20% of low quality reads were eliminated from each dataset at the filtering step. Only 25–35% of the reads which contained predicted protein coding regions (49 902–689 805 reads per sample), were taxonomically assigned using M5NR protein database. While 30–40% of reads assigned to the SEED Subsystems database were used for generation of metabolic profiles (Table 1). Each of the metagenomic datasets according to the MG-RAST statistics contained approximately 10% of reads with predicted rRNA gene fragments. The subsequent annotation revealed no reads from the AP-based dataset and only 1% of the reads from the SH- and WGA-based datasets matched the M5RNA database.

**Table 1:** General characteristics of full sequencing data.

Sequencingapproach	Average numberof reads (range)	Numberof Mbp	Average readlength, bp ± SD	FailedQC(%)	Number of reads withpredicted proteincoding regions (range)	Number of reads withpredicted rRNAgenes (range)	Number of assignedfeatures to M5NRdatabase (%)	Number of assignedfeatures to SEEDSubsystems (%)	Number of assignedfeatures to M5RNAdatabase (%)
**Shotgun**	672 542(531 108–806 483)	133.6	197±73	20	464 929(325 410–582 708)	82 151(62 899–96 886)	35	43	1.3
**APPCR**	468 187(74370–1 074 266)	70.7	142±69	24	287 840(49 902–617 609)	44 896(5 868–104 247)	26	35	0.0
**WGA**	911 553(506 028–2 012 359)	178.5	198±75	20	549 355(354 930–1 032 625)	96 117(61 694–187 539)	26	30	0.8

Statistical data represented as mean ± Standard Deviation (SD). Percentage of sequences matching to the M5NR, M5RNA and SEED Subsystems databases was determined with an E-value cut-off of E<1×10^−5^. QC = quality control.

### Taxonomic profiling of metagenomes

The analysis of metagenomic data within MG-RAST occurs both for protein coding genes and ribosomal (rRNA) genes. And therefore analysis of taxonomy can be performed in two ways.

Taxonomic classification of protein gene fragments showed that 85 (±4)% of the annotated reads were assigned to Bacteria, with 4.5 (±2.7)% of reads also matched to Eukaryota and 0.6 (±0.4)% to Archaea. The remaining 10 (±1)% of reads were not assigned. Bacterial taxa *Proteobacteria, Actinobacteria* and *Bacteroidetes* dominated in all metagenomic datasets representing close to 70% of protein annotated reads. Additional phyla including *Chloroflexi, Planctomycetes, Acidobacteria Firmicutes, Cyanobacteria, Verrucomicrobia* represented less than 5% of reads. Among the eukaryotic taxa, *Ascomicota* was found to be the dominant microorganism 3.0 (±2.6)%. Other eukaryotic taxa such as *Streptophyta, Chordata, Basidiomycota* and *Arthropoda* collectively contributed to the remaining 1% of the annotated reads (Table S2).

Taxonomic classification of the rRNA gene fragments identified only in SH- and WGA-based datasets showed that 78 (±8)% of reads were assigned to bacterial taxa and 14.5 (±6.5)% to eukaryotic taxa (data represented as an average relative abundance of taxa between the samples of SH- and WGA-based datasets). The most abundant bacterial and eukaryotic phyla found were the same as per protein-derived taxonomic classification (described above) namely: *Actinobacteria, Proteobacteria, Bacteroidetes, Ascomycota* and *Streptophyta.* The remaining 7 (±4) % of reads were not assigned (Table S3).

Rarefaction analysis was performed on randomly subsampled metagenomic datasets (49 000 reads per sample) annotated against the M5NR non-redundant protein database. The analysis showed the differences in biodiversity (highest level of taxonomic resolution) of the datasets generated by the three metagenome sequencing approaches (Fig. S1). The SH- and WGA-based datasets demonstrated a similar numbers of identified species from location A and B. A two fold lower number of species were identified in the AP-based dataset.

### Metabolic profiling of metagenomes

Metabolic profiles for all datasets were created by matching to the SEED Subsystems database. The most abundant metabolic features found in all datasets, accounting for almost 60% of assigned reads were: clustering-based subsystems; carbohydrates; amino acids and derivatives; protein metabolism; miscellaneous; cofactors; vitamins; prosthetic groups; pigments and DNA metabolism. The relative abundance each of the remaining metabolic features represented less than 5% of reads (Table S4).

### Comparison of soil metagenome profiles based on full sequence datasets

#### Comparison of protein-derived taxonomic profiles

**Figure 1:**
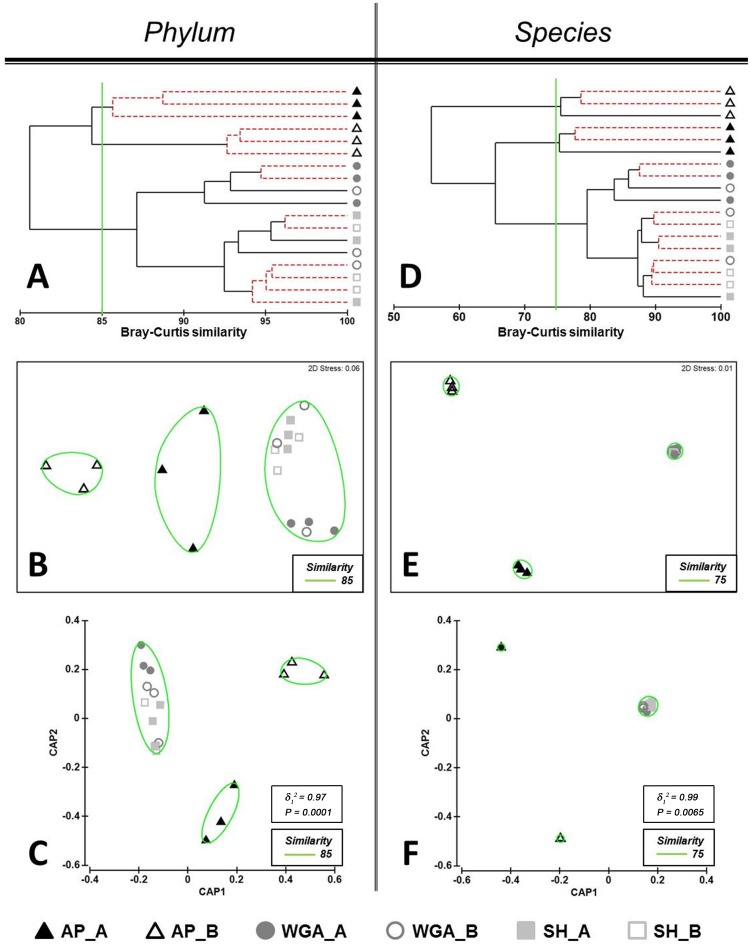
Comparison of the taxonomic soil profiles generated on full datasets at the phylum (A, B, C) and species (D, E, F) resolution levels. Bray-Curtis distance similarity matrix was calculated from the square-root transformed abundance of DNA fragments matching taxa in the M5NR database (E-value <1×10^−5^). The Bray-Curtis matrix was used for generating CLUSTER dendrogram, NMDS and CAP ordination plots. **CLUSTER analysis (A and D).** Red dotted branches on the CLUSTER dendrogram indicate no significant difference between metagenomic profiles (supported by the SIMPROF analysis, p<0.05). **NMDS unconstrained ordination (B and E).** The NMDS plot displays distances between samples. Data points that are closer to each other represent samples with highly similar metagenomic profiles. **CAP constrained ordination (C and F).** CAP analysis tests for differences among the groups in multivariate space. The significance of group separation along the canonical axis is indicated by the value of the squared canonical correlation (δ_1_^2^) and P-value. A contour line on the NMDS and CAP ordinations drawn round each of the cluster defines the superimposition of clusters from CLUSTER dendrogram at the selected level of similarity.

An initial comparison of the taxonomic structures of the metagenomes using lowest (coarsest) resolution profiles derived at the phylum level of taxonomy was performed. CLUSTER analysis with group-average linking based on Bray-Curtis similarity matrices delineated two distinct clusters with similarity of 85% formed by samples from AP-based dataset grouped according to the sites from where the samples were taken (Fig. 1A). These clusters were supported by the SIMPROF analysis that showed statistically significant (p<0.05) evidence of genuine clustering, as indicated by red dotted branches on the dendrogram (Fig. 1A). Two samples from WGA_A group having 94% profiles similarity also formed such a cluster. Other samples form SH- and WGA-based datasets formed mixed clusters. For example, a sample from the WGA_B group formed a united cluster with a sample from the SH_A group and two samples from the SH_B group (similarity 94%), thus indicating that the samples from two different locations were grouped together incorrectly. One more cluster consisted of two samples from SH_A and SH_B groups with 96% of similarity.

Bray-Curtis distances between metagenomic profiles were then displayed on an NMDS plot (Fig. 1B). NMDS analysis did not reveal a clear visual separation of data. Points denoting samples from WGA- and SH-based datasets were located much closer together showing a higher similarity of the profiles than points representing AP-based dataset (Fig. 1B). Overlaying clusters on the NMDS plot made visual discrimination of the patterns formed by AP-based dataset easier (Fig. 1B).

It has been noted that the distinct patterns of multi-dimensional datasets could be hidden in the low-dimensional space of NMDS ordination [46]. Consequently for the comparison of our metagenomics datasets, CAP analysis as a constrained ordination method was also performed. CAP analysis tests the hypothesis of whether there is a difference between pre-defined groups. In our research all datasets were divided into 6 groups in accordance with combined factors, including the sequencing approach applied and the origin of soil samples. The results of the CAP ordination at the phylum level demonstrated that the first squared canonical correlation was very large (δ_1_^2^ = 0.97), indicating the significance of the CAP model. The first canonical axis showed clear separation of the samples within AP-based dataset according to the soil sampling sites. At the same time a close overlapping of the samples from the SH- and WGA-based datasets was observed (Fig. 1C). However, the cross-validation results of the CAP model for the chosen value of *m* = 6 did not confirm the above defined separation of the metagenomic datasets (Table 2). Thus, the most distinct groups, which had a 100% success under cross-validation, were AP_B and WGA_A. One sample from the AP_A group was misclassified to the AP_B group. One sample from each of the SH_A and SH_B groups were misclassified to the SH_B and SH_A groups, respectively. All the samples from the WGA_B group were misclassified to another three different groups (SH_A, SH_B and WGA_A).

**Table 2:** Results of CAP model cross-validation of soil taxonomic profiles discrimination generated from full sequencing datasets.

Original Group		AP_A	AP_B	WGA_A	WGA_B	SH_A	SH_B
Taxonomy level	***phylum*** (*m* = 6, δ_1_^2^ = 0.97, P = 0.0001)						
% correct		67	100	100	0	67	67
correct/total		2/3	3/3	3/3	0/3	2/3	2/3
Misclassifiedto group		AP_B	n/a	n/a	SH_A	SH_B	SH_A
					SH_B		
					WGA_A		
Taxonomy level	***class*** (*m* = 5, δ_1_^2^ = 0.98, P = 0.0001)						
% correct		100	100	100	0	67	33
correct/total		3/3	3/3	3/3	0/3	2/3	1/3
Misclassified togroup					SH_B		SH_A
		n/a	n/a	n/a	WGA_A	WGA_B	WGA_B
					SH_A		
Taxonomy level	***order*** (*m* = 3, δ_1_^2^ = 0.97, P = 0.0002)						
% correct		100	100	100	0	67	33
correct/total		3/3	3/3	3/3	0/3	2/3	1/3
Misclassified togroup					SH_B		SH_A
		n/a	n/a	n/a	WGA_A	SH_B	WGA_B
					SH_A		
Taxonomy level	***family*** (*m* = 10, δ_1_^2^ = 0.99, P = 0.0034)						
% correct		100	100	100	0	67	67
correct/total		3/3	3/3	3/3	0/3	2/3	2/3
Misclassified togroup					SH_B		
		n/a	n/a	n/a	WGA_A	SH_B	WGA_B
					SH_A		
Taxonomy level	***genus*** (*m* = 11, δ_1_^2^ = 0.99, P = 0.01)						
% correct		100	100	100	0	100	67
correct/total		3/3	3/3	3/3	0/3	3/3	2/3
Misclassified togroup					SH_B		
		n/a	n/a	n/a	WGA_A	n/a	WGA_B
					SH_A		
Taxonomy level	***species*** (*m* = 10, δ_1_^2^ = 0.99, P = 0.0065)						
% correct		100	100	100	0	67	67
correct/total		3/3	3/3	3/3	0/3	2/3	2/3
Misclassified togroup					SH_B		
		n/a	n/a	n/a	WGA_A	SH_B	WGA_B
					SH_A		

It is of note that apart from AP_A and WGA_A groups at the class level of taxonomic resolution the cross-validation of the CAP model showed a 100% correct classification of the samples from AP_B group (Table 2). Additionally one sample from the SH_A group was misclassified to the WGA_B group, whereas two samples from the SH_B group were misclassified to the SH_A and WGA_B groups.

Further CLUSTER analysis, NMDS and CAP ordinations of the metagenomic samples at higher levels of taxonomy demonstrated similar patterns of differentiation as observed at the phylum and class levels (Figs.1, S2,S3). Thus, at the order, family, genus and species levels of resolution two samples from the WGA_A group and two samples from the SH_A group formed separate genuine clusters on the CLUSTER dendrograms (Fig. 1D, S2D, S3). Two more genuine mixed clusters were observed consisting of the samples from the SH_B and the WGA_B groups. NMDS and CAP ordinations at all levels of resolution clearly displayed three distinct clusters; two clusters consisting of the samples from the AP_A and the AP_B groups and one mixed cluster of samples from all the other groups (Fig. 1, S2, S3). Cross-validation results of the CAP models at all levels of resolution, starting from the class level, showed an accurate 100% correct classification of samples from the AP-based dataset (Table 2). Despite the visual overlapping of the SH- and WGA-based data points shown on the ordination plots (Fig. 1, S2, S3), the samples from WGA_A group were classified 100% correctly across all levels of taxonomic resolution (Table 2). Of note was that, at the genus level, all samples from the SH_A group were also successfully allocated.

#### Comparison of taxonomic profiles based on rRNA gene fragment classification

Taxonomic profiles were generated only for the SH- and the WGA-based datasets where the rRNA gene fragments matched to the M5RNA database. The AP-based dataset was excluded from the consecutive comparative analysis since no sequence matches to the ribosomal database were found. CLUSTER analysis of rRNA-based taxonomic profiles at the phylum level of resolution demonstrated the formation of four genuine clusters confirmed by SIMPROF analysis (p<0.05) (Fig. S4A). One cluster included three samples from the WGA_A group and one sample from the WGA_B group with similarity of 77%. A second cluster consisted of two samples from the SH_A group and one sample from the SH_B group with similarity of 85%. Two other mixed clusters were formed by the samples from different groups. The pattern formed by the samples from the WGA_A group was also seen on the NMDS and CAP plots with a 100% correct allocation which was confirmed by the results of cross-validation of the CAP model (Fig. S4B, S4C; Table S7). Two separate clusters formed by the samples from the WGA_A and the SH_A groups were observed at the higher levels of taxonomic resolution (genus and species) (Figs. S6). Observed groupings had a 100% correct allocation under cross-validation of the CAP model only at the genus level of classification (Table S7). The latter findings were in full accordance with the allocation of WGA_A and SH_A groups performed using protein-derived taxonomy (Table 2).

#### Metabolic profiles comparison

**Figure 2:**
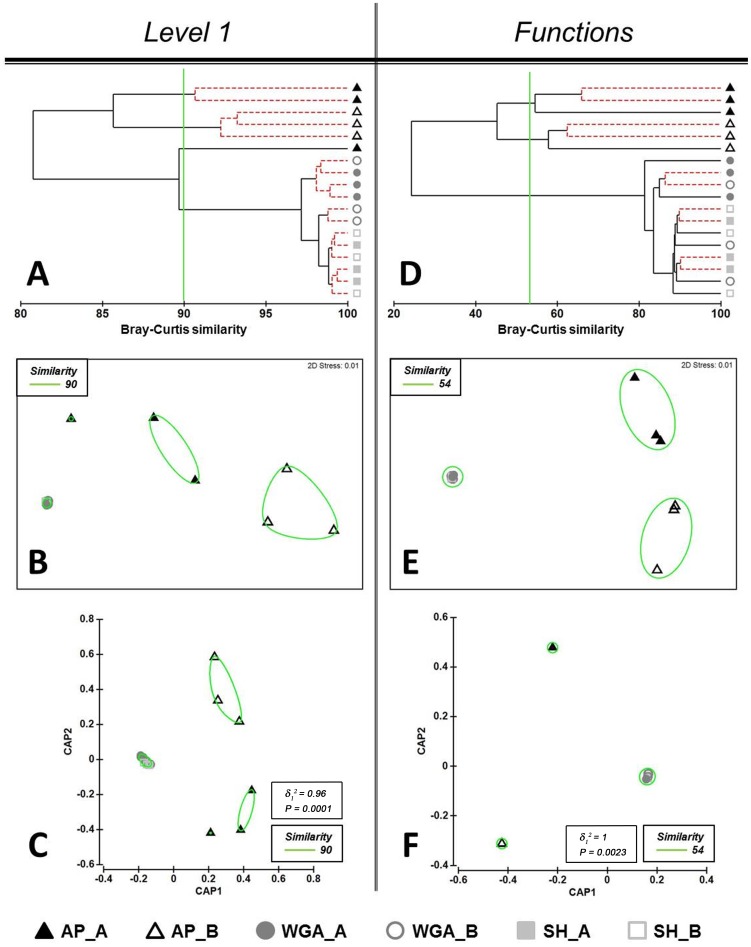
Comparison of the metabolic soil profiles generated on full datasets at the subsystems level 1 (A, B, C) and subsystems function (D, E, F) resolution levels. Bray-Curtis distance similarity matrix was calculated from the square-root transformed abundance of DNA fragments matching taxa in the SEED database (E-value <1×10^−5^). The Bray-Curtis matrix was used for generating CLUSTER dendrogram, NMDS and CAP ordination plots. **CLUSTER analysis (A and D).** Red dotted branches on the CLUSTER dendrogram indicate no significant difference between metagenomic profiles (supported by the SIMPROF analysis, p<0.05). **NMDS unconstrained ordination (B and E).** The NMDS plot displays distances between samples. Data points that are closer to each other represent samples with highly similar metagenomic profiles. **CAP constrained ordination (C and F).** CAP analysis tests for differences among the groups in multivariate space. The significance of group separation along the canonical axis is indicated by the value of the squared canonical correlation (δ_1_^2^) and P-value. A contour line on the NMDS and CAP ordinations drawn round each of the cluster defines the superimposition of clusters from CLUSTER dendrogram at the selected level of similarity.

CLUSTER analysis of metabolic profiles generated by different sequencing approaches at the lowest level of resolution (level 1) showed that all three samples from the AP_B group formed a separate cluster with a similarity of 92% (Fig. 2A). Two samples from the AP_A group had a similarity of 90%. The third AP_A sample was bundled with the samples from SH- and WGA- based datasets forming a new mixed cluster. Importantly the SH- and WGA-based datasets consisting of 12 metagenomic samples formed one united mixed cluster with a similarity of 97% (Fig. 2A). NMDS and CAP ordinations also showed that all the points associated with the samples from SH- and WGA-based datasets produced a very compact cluster (Fig. 2B and Fig. 2C). However, according to a cross-validation procedure the most distinct groups with 100% allocation success were the AP-based groups and the WGA_A group, whereas misclassification errors were shown for the WGA_B, SH_A and SH_B groups (Table 3). Statistical comparisons of the metabolic profiles at higher resolution levels (level 2, level 3 and function) resulted in similar discriminating success (Fig. 2, S7). CLUSTER analysis showed correct site-specific grouping of the samples from AP-based dataset (Fig. 2D, S7). All the profiles produced by SH- and WGA-based methods again formed a single unresolved cluster. NMDS and CAP ordinations demonstrated clear separation of three clusters (Fig. 2, S7), which was also the case for the metagenomic profiles comparison based on protein-derived taxonomy (Fig. 1, S2, S3). In both cases cross-validation results of the CAP model gave 100% correct classification of the samples from the AP_A, AP_B and WGA_A groups and misclassification errors for samples from the SH_A, SH_B and WGA_B groups (Table 2, Table 3).

**Table 3:** Results of CAP model cross-validation of soil metabolic profiles discrimination generated from full sequencing datasets.

Original Group		AP_A	AP_B	WGA_A	WGA_B	SH_A	SH_B
Metabolic level	***level 1*** (*m* = 2, δ_1_^2^ = 0.96, P = 0.0001)						
% correct		100	100	100	33	67	33
correct/total		3/3	3/3	3/3	1/3	2/3	1/3
Misclassified togroup		n/a	n/a	n/a	SH_A	SH_B	SH_A
					SH_B		WGA_B
Metabolic level	***level 2*** (*m* = 11, δ_1_^2^ = 1, P = 0.0002)						
% correct		100	100	100	33	67	100
correct/total		3/3	3/3	3/3	1/3	2/3	3/3
Misclassified togroup					SH_B		
		n/a	n/a	n/a	WGA_A	SH_B	n/a
Metabolic level	***level 3*** (*m* = 12, δ_1_^2^ = 1, P = 0.0009)						
% correct		100	100	100	33	67	67
correct/total		3/3	3/3	3/3	1/3	2/3	2/3
Misclassified togroup					SH_B		
		n/a	n/a	n/a	WGA_A	SH_B	SH_A
Metabolic level	***functions*** (*m* = 10, δ_1_^2^ = 1, P = 0.0023)						
% correct		100	100	67	0	67	67
correct/total		3/3	3/3	2/3	0/3	2/3	2/3
Misclassified togroup					SH_B		
		n/a	n/a	WGA_B	WGA_A	SH_B	SH_A
					SH_B		

### Comparison of metagenomic profiles based on randomly sub-sampled datasets

#### Comparison of taxonomic profiles based on rRNA gene fragment classification

CLUSTER analysis and NMDS ordination of rRNA-based taxonomic profiles at the phylum level of taxonomy demonstrated a heterogeneous mixed cluster of the samples from the SH- and WGA-based datasets with an average similarity of 70% (data not shown). Cap analysis showed 100% correct classification of samples from the WGA_A group and misclassification errors for samples from other groups. At the species level of resolution CLUSTER analysis also revealed a single heterogeneous mixed cluster with thea taxonomic profile similarity of approximately 25%. CAP analysis indicated a high degree of misclassification errors.

#### Comparison of protein-derived taxonomic and metabolic profiles

It has been proposed that in order to enable the comparison of metagenomes based on equal sequencing efforts, the datasets should be randomly sub-sampled to the size of the smallest sample [17], [47]. The metagenomic datasets generated by shotgun, WGA-based and AP-PCR-based approaches were re-analysed by MG-RAST at an equivalent sequencing depth of 49 000 annotated reads per sample. Comparison of taxonomic and metabolic profiles generated from sub-sampled datasets at all levels of classification available within MG-RAST was performed by CLUSTER analysis, NMDS and CAP ordination.

Statistical analysis of the sub-sampled metagenomic datasets generated by three metagenome sequencing approaches yielded nearly identical estimates of the overall differences between soil microbial communities from locations A and B as those obtained using full sequence datasets (Figs. S8– S12, Tables S5– S6). This similarity was also confirmed using the RELATE programme which revealed a strong correlation between Bray-Curtis distance matrices (Spearman rank coefficient r>0.9, p<0.0001) generated on both full, and sub-sampled, datasets at all levels of taxonomic and metabolic resolution (Table 4).

**Table 4:** RELATE comparison of Bray-Curtis similarity matrices.

Taxonomic level	Spearman rank coefficient	Metabolic level	Spearman rank coefficient
phylum	0.887	level 1	0.652
class	0.944	level 2	0.958
order	0.959	level 3	0.967
family	0.940	functions	0.969
genus	0.965		
species	0.966		

The Bray-Curtis similarity matrices calculated from square root transformed abundance of DNA fragments generated based on full datasets and sub-sampled datasets.

## Discussion

Numerous ecological studies show that soil microbial communities differ between land uses and vegetation types [17], [18], [33], [48], [49]. The discrimination of geographically distinct urban soils with similar land management type and similar plant cover is of great forensic relevance [7], [34]. If two soil samples appear very different visually then a simple exclusion can be made but more typically soils appear visually similar and currently no further action is taken. The vast majority of samples submitted for forensic investigation come from urban areas; here we include gardens, parkland and open spaces as well as built-up areas. Thus we focused our study on assessing the ability of three random whole metagenomic sequencing approaches to describe and differentiate the composition of soil microbial communities from two random parklands in 3 km apart within Adelaide residential areas. The vegetation categories of these locations appeared to be very similar, with widespread grass and trees species.

Along with standard metagenomic approaches such as shotgun and WGA, which are widely accepted as the most comprehensive sources of data for studying complex microbial communities, we evaluated AP-PCR as a method for generation of random metagenomic DNA profiles of soils that were then analysed by high throughput DNA sequencing. In this technique an arbitrary chosen oligonucleotide is used as a single primer that targets multiple genomic sequences producing a highly primer-sequence-specific profiles. Depending both on the primer chosen and the annealing temperature used there is sequence specific selection of complementary sequences to the primer to be DNA amplified. Based on previous studies the random amplification of polymorphic DNA, normally performed at low stringency conditions (low annealing temperature), becomes more reproducible at high stringency amplification conditions [50]. Amplification with a single arbitrary primer yields an arbitrary product pattern which might possess PCR products from both abundant species and those that are rare, again depending on the affinity of the primer.

The composition of the soil microbial communities was determined from both taxonomic classification of rRNA fragments and the taxonomic assignment of functional gene fragments. Similar taxonomic distribution of dominant microbial phyla was observed across all metagenomic datasets using these two different annotation pipelines. Reads with functional gene fragments were also used for the comparison of metagenomic datasets based on metabolic profiles.

Previous reports have indicated that comparison of metagenomes at low levels of resolution, i.e. analysis based on more broadly defined categories, results in a more conservative estimate of the distances between metagenomic profiles [17]. Low levels of taxonomic or functional classification show less overlap between samples and are therefore also used frequently for metagenomic profile discrimination [51], [52]. The results of the metagenomic dataset comparison in the current study are presented at all MG-RAST taxonomic (phylum to species) and metabolic (level 1 to functions) levels of hierarchy. The comparison of metagenomes was performed with a number of unconstrained statistical tools including CLUSTER and NMDS analyses as well as constrained CAP analysis testing a predefined hypothesis that was previously shown to be successful for soil microbial communities discrimination [46], [53].

SH- and WGA-based metagemonic sequencing approaches showed incorrect and inconsistent discrimination of soil samples according to sampling sites using both taxonomic (protein and ribosomal) and metabolic classifications. Comparison of the SH- and WGA-based profiles revealed not only misclassification of the samples between the locations but often between repeat analysis of each sequencing approach, with the exception of the WGA_A samples which had a 100% allocation success. The high similarity of the data generated by these methods appears to be driven by the highly similar, or even identical, dominant microorganisms found in the soil samples collected from two distinct sites of similar urban type. This supports the theory that the data generated by shotgun sequencing are commonly shifted towards describing the most abundant taxa leaving the contribution of rare microorganisms undervalued for comparative analysis [54].

A rarefaction analysis was performed to determine microbial species richness of metagenomic datasets produced by three random whole metagenome sequencing approaches for the soil samples from location A and B. The rarefaction curves computed for metagenomic datasets did not reach the plateau phase suggesting that more sequencing effort would be required to achieve species saturation. At the same time the analysis showed that the SH- and WGA-based approaches provided a higher number of species from the same number of sequence reads than the AP-based approach. The AP-PCR utilises primer dependant sequence specific selection of gene fragments and therefore unlikely to amplify all the DNA fragments present in samples. Nevertheless, despite the lower species coverage of soil metagenomes provided by the AP-based approach it allowed for a 100% correct discrimination between soils samples from different locations. This may be as a result of the pre-enrichment mechanisms of AP-PCR that are based on the primer sequence targeting both dominant and rare microorganisms equally. An AP-PCR-based strategy for whole metagenomic profile generation may be compromised by artefacts, including chimeric sequences caused by PCR amplification, which have been reported for gene-targeted (e.g. 16S) sequencing approaches [19]. It is likely that the AP-PCR based approach does not reflect the true picture of the soil microbial community composition. However, we found consistent evidence that an AP-PCR-based whole metagenome sequencing approach was able to discriminate similar soil samples based on differences in both taxonomic and metabolic compositions.

## Conclusion

In the research presented here we investigated the ability of whole metagenome analysis techniques to discriminate soil samples of similar land use and vegetation type but collected from different geographical locations. There is currently no agreed evaluation approach leading to an accurate picture of the soil metagenome structure as the true soil microbial community composition [55]. Three methods of whole soil metagenome analysis based on high-throughput DNA sequencing were assessed; shotgun, whole genome amplification and arbitrarily primed PCR. The metagenomic datasets underwent comprehensive statistical analysis using unconstrained and constrained approaches including CLUSTER analysis, NMDS and CAP ordination at all levels of both taxonomic and metabolic classification. The shotgun and WGA-based approaches generated highly similar metagenomic profiles for soil samples such that the soil samples could not be distinguished. An AP-PCR-based approach was shown to be the most powerful technique for obtaining site-specific metagenomic DNA profiles which were able to successfully discriminate between similar soil samples taken from different locations.

The methods presented in this study show a significant step towards possible implementation of forensic soil discrimination using random whole metagenomics for investigation and evidence generation. By increasing the amount of samples analysed from each location and also by increasing the number of distinct geographical locations it will become possible to train algorithms that can then be used for comparison to unknown soil samples obtained as part of criminal investigations. The power of discrimination of these tools is proportional to the amount of samples taken and ultimately the unique metagenomic profile of the different locations. The investigation of temporal microbial variation would further strengthen any tool that is developed. As the sample sizes increase the tool will move from the model developed in this study to one that has sufficient power as a useful investigative tool and ultimately to a method that can be presented in court. The step to being a useful investigative tool for law enforcement can be made from the current study with increased repetition and geographic sampling. For presentation in a court of law the development of a sufficient sample size and distinct geographic profiles will need to be bolstered with a determination of the limitations of the method, including false positive and negative rates. This can be achieved via blind trials, mock case work and a period of casework hardening in order to achieve the levels require for acceptance.

## Supporting Information

Figure S1 **Rarefaction curves created in MG-RAST.** Rarefaction analysis was performed at the species level for each metagenomic protein-derived taxonomic profile based on randomly sub-samples datasets (49 000 reads per sample). The curves for all taxa include Bacteria, Archaea, Eukaryota, Viruses, unclassified and other sequences identified after metagenomic dataset annotation with M5NR database.
(TIF)

Figure S2 **Comparison of the soil protein-derived taxonomic profiles generated on full datasets at the class (A, B, C) and order (D, E, F) taxonomic resolution levels.** Bray-Curtis distance similarity matrix was calculated from the square-root transformed abundance of DNA fragments matching taxa to the M5NR database (E-value <1×10^−5^). The Bray-Curtis matrix was used for generating CLUSTER dendrogram, NMDS and CAP ordination plots. **CLUSTER analysis (A and D)**. Red dotted branches on the CLUSTER dendrogram indicate no significant difference between metagenomic profiles (supported by the SIMPROF analysis, p<0.05). **NMDS unconstrained ordination (B and E)**. The NMDS plot displays distances between samples. Data points that are closer to each other represent samples with highly similar metagenomic profiles. **CAP constrained ordination (C and F)**. CAP analysis tests for differences among the groups in multivariate space. The significance of group separation along the canonical axis is indicated by the value of the squared canonical correlation (δ_1_^2^) and P-value (P<0.05). A contour line on the NMDS and CAP ordinations drawn round each of the cluster defines the superimposition of clusters from CLUSTER dendrogram at the selected level of similarity.
(TIF)

Figure S3 **Comparison of the soil rRNA profiles generated on full datasets at the phylum (A, B, C) and class (D, E, F) taxonomic resolution levels.** Bray-Curtis distance similarity matrix was calculated from the square-root transformed abundance of DNA fragments matching taxa in the M5RNA database (E-value <1×10^−5^). The Bray-Curtis matrix was used for generating CLUSTER dendrogram, NMDS and CAP ordination plots. **CLUSTER analysis (A and D)**. Red dotted branches on the CLUSTER dendrogram indicate no significant difference between metagenomic profiles (supported by the SIMPROF analysis, p<0.05). **NMDS unconstrained ordination (B and E)**. The NMDS plot displays distances between samples. Data points that are closer to each other represent samples with highly similar metagenomic profiles. **CAP constrained ordination (C and F)**. CAP analysis tests for differences among the groups in multivariate space. The significance of group separation along the canonical axis is indicated by the value of the squared canonical correlation (δ_1_^2^) and P-value (P<0.05). A contour line on the NMDS and CAP ordinations drawn round each of the cluster defines the superimposition of clusters from CLUSTER dendrogram at the selected level of similarity.
(TIF)

Figure S4 **Comparison of the soil rRNA profiles generated on full datasets at the phylum (A, B, C) and class (D, E, F) taxonomic resolution levels.** Bray-Curtis distance similarity matrix was calculated from the square-root transformed abundance of DNA fragments matching taxa in the M5RNA database (E-value <1×10^−5^). The Bray-Curtis matrix was used for generating CLUSTER dendrogram, NMDS and CAP ordination plots. **CLUSTER analysis (A and D)**. Red dotted branches on the CLUSTER dendrogram indicate no significant difference between metagenomic profiles (supported by the SIMPROF analysis, p<0.05). **NMDS unconstrained ordination (B and E)**. The NMDS plot displays distances between samples. Data points that are closer to each other represent samples with highly similar metagenomic profiles. **CAP constrained ordination (C and F)**. CAP analysis tests for differences among the groups in multivariate space. The significance of group separation along the canonical axis is indicated by the value of the squared canonical correlation (δ_1_^2^) and P-value (P<0.05). A contour line on the NMDS and CAP ordinations drawn round each of the cluster defines the superimposition of clusters from CLUSTER dendrogram at the selected level of similarity.
(TIF)

Figure S5 **Comparison of the soil rRNA profiles generated on full datasets at the order (A, B, C) and family (D, E, F) taxonomic resolution levels.** Bray-Curtis distance similarity matrix was calculated from the square-root transformed abundance of DNA fragments matching taxa in the M5RNA database (E-value <1×10^−5^). The Bray-Curtis matrix was used for generating CLUSTER dendrogram, NMDS and CAP ordination plots. **CLUSTER analysis (A and D)**. Red dotted branches on the CLUSTER dendrogram indicate no significant difference between metagenomic profiles (supported by the SIMPROF analysis, p<0.05). **NMDS unconstrained ordination (B and E)**. The NMDS plot displays distances between samples. Data points that are closer to each other represent samples with highly similar metagenomic profiles. **CAP constrained ordination (C and F)**. CAP analysis tests for differences among the groups in multivariate space. The significance of group separation along the canonical axis is indicated by the value of the squared canonical correlation (δ_1_^2^) and P-value (P<0.05). A contour line on the NMDS and CAP ordinations drawn round each of the cluster defines the superimposition of clusters from CLUSTER dendrogram at the selected level of similarity.
(TIF)

Figure S6 **Comparison of the soil rRNA profiles generated on full datasets at the genus (A, B, C) and species (D, E, F) taxonomic resolution levels.** Bray-Curtis distance similarity matrix was calculated from the square-root transformed abundance of DNA fragments matching taxa in the M5RNA database (E-value <1×10^−5^). The Bray-Curtis matrix was used for generating CLUSTER dendrogram, NMDS and CAP ordination plots. **CLUSTER analysis (A and D)**. Red dotted branches on the CLUSTER dendrogram indicate no significant difference between metagenomic profiles (supported by the SIMPROF analysis, p<0.05). **NMDS unconstrained ordination (B and E)**. The NMDS plot displays distances between samples. Data points that are closer to each other represent samples with highly similar metagenomic profiles. **CAP constrained ordination (C and F)**. CAP analysis tests for differences among the groups in multivariate space. The significance of group separation along the canonical axis is indicated by the value of the squared canonical correlation (δ_1_^2^) and P-value (P<0.05). A contour line on the NMDS and CAP ordinations drawn round each of the cluster defines the superimposition of clusters from CLUSTER dendrogram at the selected level of similarity.
(TIF)

Figure S7 **Comparison of the soil metabolic profiles generated on full datasets at the subsystems level 2 (A, B, C) and level 3 (D, E, F) metabolic resolution levels.** Bray-Curtis distance similarity matrix was calculated from the square-root transformed abundance of DNA fragments matching taxa in the SEED database (E-value <1×10^−5^). The Bray-Curtis matrix was used for generating CLUSTER dendrogram, NMDS and CAP ordination plots. **CLUSTER analysis (A and D)**. Red dotted branches on the CLUSTER dendrogram indicate no significant difference between metagenomic profiles (supported by the SIMPROF analysis, p<0.05). **NMDS unconstrained ordination (B and E)**. The NMDS plot displays distances between samples. Data points that are closer to each other represent samples with highly similar metagenomic profiles. **CAP constrained ordination (C and F)**. CAP analysis tests for differences among the groups in multivariate space. The significance of group separation along the canonical axis is indicated by the value of the squared canonical correlation (δ_1_^2^) and P-value (P<0.05). A contour line on the NMDS and CAP ordinations drawn round each of the cluster defines the superimposition of clusters from CLUSTER dendrogram at the selected level of similarity.
(TIF)

Figure S8 **Comparison of the soil protein-derived taxonomic profiles generated on randomly sub-sampled datasets at the phylum (A, B, C) and class (D, E, F) metabolic resolution levels.** Bray-Curtis distance similarity matrix was calculated from the square-root transformed abundance of DNA fragments matching taxa in the M5NR database (E-value <1×10^−5^). The Bray-Curtis matrix was used for generating CLUSTER dendrogram, NMDS and CAP ordination plots. **CLUSTER analysis (A and D)**. Red dotted branches on the CLUSTER dendrogram indicate no significant difference between metagenomic profiles (supported by the SIMPROF analysis, p<0.05). **NMDS unconstrained ordination (B and E)**. The NMDS plot displays distances between samples. Data points that are closer to each other represent samples with highly similar metagenomic profiles. **CAP constrained ordination (C and F)**. CAP analysis tests for differences among the groups in multivariate space. The significance of group separation along the canonical axis is indicated by the value of the squared canonical correlation (δ_1_^2^) and P-value (P<0.05). A contour line on the NMDS and CAP ordinations drawn round each of the cluster defines the superimposition of clusters from CLUSTER dendrogram at the selected level of similarity.
(TIF)

Figure S9 **Comparison of the soil protein-derived taxonomic profiles generated on randomly sub-sampled datasets at the order (A, B, C) and family (D, E, F) taxonomic resolution levels.** Bray-Curtis distance similarity matrix was calculated from the square-root transformed abundance of DNA fragments matching taxa in the SEED database (E-value <1×10^−5^). The Bray-Curtis matrix was used for generating CLUSTER dendrogram, NMDS and CAP ordination plots. **CLUSTER analysis (A and D)**. Red dotted branches on the CLUSTER dendrogram indicate no significant difference between metagenomic profiles (supported by the SIMPROF analysis, p<0.05). **NMDS unconstrained ordination (B and E)**. The NMDS plot displays distances between samples. Data points that are closer to each other represent samples with highly similar metagenomic profiles. **CAP constrained ordination (C and F)**. CAP analysis tests for differences among the groups in multivariate space. The significance of group separation along the canonical axis is indicated by the value of the squared canonical correlation (δ_1_^2^) and P-value (P<0.05). A contour line on the NMDS and CAP ordinations drawn round each of the cluster defines the superimposition of clusters from CLUSTER dendrogram at the selected level of similarity.
(TIF)

Figure S10 **Comparison of the soil protein-derived taxonomic profiles generated on randomly sub-sampled datasets at the genus (A, B, C) and species (D, E, F) taxonomic resolution levels.** Bray-Curtis distance similarity matrix was calculated from the square-root transformed abundance of DNA fragments matching taxa in the M5NR database (E-value <1×10^−5^). The Bray-Curtis matrix was used for generating CLUSTER dendrogram, NMDS and CAP ordination plots. **CLUSTER analysis (A and D)**. Red dotted branches on the CLUSTER dendrogram indicate no significant difference between metagenomic profiles (supported by the SIMPROF analysis, p<0.05). **NMDS unconstrained ordination (B and E)**. The NMDS plot displays distances between samples. Data points that are closer to each other represent samples with highly similar metagenomic profiles. **CAP constrained ordination (C and F)**. CAP analysis tests for differences among the groups in multivariate space. The significance of group separation along the canonical axis is indicated by the value of the squared canonical correlation (δ_1_^2^) and P-value (P<0.05). A contour line on the NMDS and CAP ordinations drawn round each of the cluster defines the superimposition of clusters from CLUSTER dendrogram at the selected level of similarity.
(TIF)

Figure S11 **Comparison of the soil metabolic profiles generated on randomly sub-sampled datasets at the subsystems level 1 (A, B, C) and subsystems Level 2 (D, E, F) metabolic resolution levels.** Bray-Curtis distance similarity matrix was calculated from the square-root transformed abundance of DNA fragments matching taxa in the SEED database (E-value <1×10^−5^). The Bray-Curtis matrix was used for generating CLUSTER dendrogram, NMDS and CAP ordination plots. **CLUSTER analysis (A and D)**. Red dotted branches on the CLUSTER dendrogram indicate no significant difference between metagenomic profiles (supported by the SIMPROF analysis, p<0.05). **NMDS unconstrained ordination (B and E)**. The NMDS plot displays distances between samples. Data points that are closer to each other represent samples with highly similar metagenomic profiles. **CAP constrained ordination (C and F)**. CAP analysis tests for differences among the groups in multivariate space. The significance of group separation along the canonical axis is indicated by the value of the squared canonical correlation (δ_1_^2^) and P-value (P<0.05)… A contour line on the NMDS and CAP ordinations drawn round each of the cluster defines the superimposition of clusters from CLUSTER dendrogram at the selected level of similarity.
(TIF)

Figure S12 **Comparison of the soil metabolic profiles generated on randomly sub-sampled datasets at the subsystems level 3 (A, B, C) and subsystems functions (D, E, F) metabolic resolution levels.** Bray-Curtis distance similarity matrix was calculated from the square-root transformed abundance of DNA fragments matching taxa in the SEED database (E-value <1×10^−5^). The Bray-Curtis matrix was used for generating CLUSTER dendrogram, NMDS and CAP ordination plots. **CLUSTER analysis (A and D)**. Red dotted branches on the CLUSTER dendrogram indicate no significant difference between metagenomic profiles (supported by the SIMPROF analysis, p<0.05). **NMDS unconstrained ordination (B and E)**. The NMDS plot displays distances between samples. Data points that are closer to each other represent samples with highly similar metagenomic profiles. **CAP constrained ordination (C and F)**. CAP analysis tests for differences among the groups in multivariate space. The significance of group separation along the canonical axis is indicated by the value of the squared canonical correlation (δ_1_^2^) and P-value (P<0.05). A contour line on the NMDS and CAP ordinations drawn round each of the cluster defines the superimposition of clusters from CLUSTER dendrogram at the selected level of similarity.
(TIF)

Table S1 **Summary of soil metagenomic samples.** All metagenomes are publically available on the MG-RAST server (http://metagenomics.anl.gov/).
(PDF)

Table S2 **Protein-derived taxonomic composition of the soil microbial communities.** Relative abundances of major taxa (phylum level) derived from taxonomic assignment of protein gene fragments matched to M5NR database.
(PDF)

Table S3 **Taxonomic composition of the soil microbial communities based on rRNA gene fragments classification.** Relative abundances of major taxa (phylum level) derived from taxonomic assignment of rRNA gene fragments matched to M5RNA database.
(PDF)

Table S4 **Metabolic composition of the soil microbial communities.** Relative abundances of major metabolic features (level 1) derived from annotation of protein gene fragments to SEED Subsystems database.
(PDF)

Table S5 **Results of CAP model cross-validation of soil protein-derived taxonomic profiles discrimination generated from sub-sampled sequencing datasets.**
(PDF)

Table S6 **Results of CAP model cross-validation of soil metabolic profiles discrimination generated from sub-sampled sequencing datasets.**
(PDF)

Table S7 **Results of CAP model cross-validation of soil rRNA taxonomic profiles discrimination generated from full sequencing datasets.**
(PDF)
