# Supplementary figures and images for: Random Whole Metagenomic Sequencing for Forensic Discrimination of Soils
Source: PLoS One. 2014 Aug 11;9(8):e104996. doi: 10.1371/journal.pone.0104996 (PMC4128759; doi:10.1371/journal.pone.0104996)

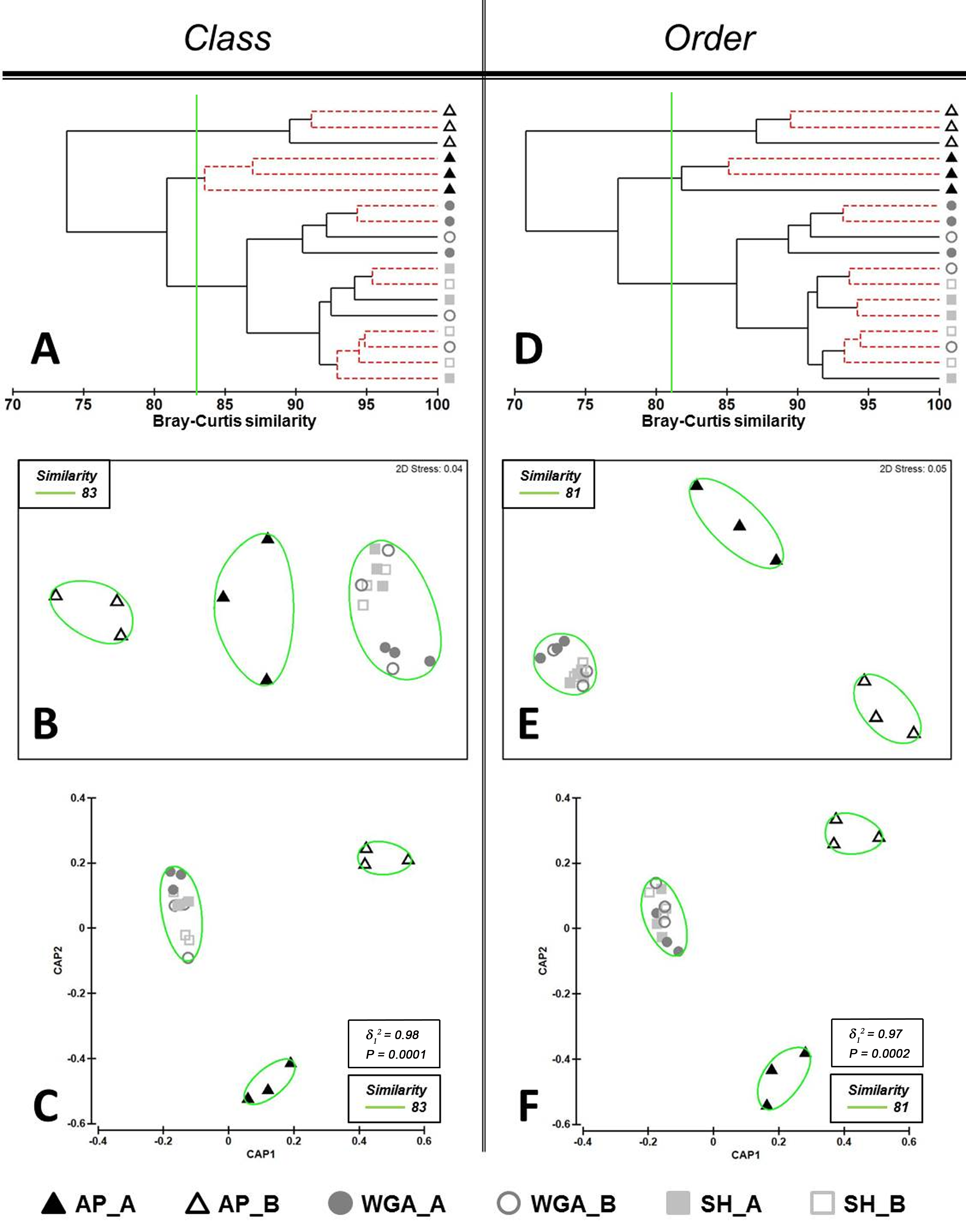

Supplement: Figure S2 — Comparison of the soil protein-derived taxonomic profiles generated on full datasets at the class (A, B, C) and order (D, E, F) taxonomic resolution levels. Bray-Curtis distance similarity matrix was calculated from the square-root transformed abundance of DNA fragments matching taxa to the M5NR database (E-value <1×10^−5^). The Bray-Curtis matrix was used for generating CLUSTER dendrogram, NMDS and CAP ordination plots. CLUSTER analysis (A and D). Red dotted branches on the CLUSTER dendrogram indicate no significant difference between metagenomic profiles (supported by the SIMPROF analysis, p<0.05). NMDS unconstrained ordination (B and E). The NMDS plot displays distances between samples. Data points that are closer to each other represent samples with highly similar metagenomic profiles. CAP constrained ordination (C and F). CAP analysis tests for differences among the groups in multivariate space. The significance of group separation along the canonical axis is indicated by the value of the squared canonical correlation (δ1 2) and P-value (P<0.05). A contour line on the NMDS and CAP ordinations drawn round each of the cluster defines the superimposition of clusters from CLUSTER dendrogram at the selected level of similarity. (TIF) [file pone.0104996.s002.tif]

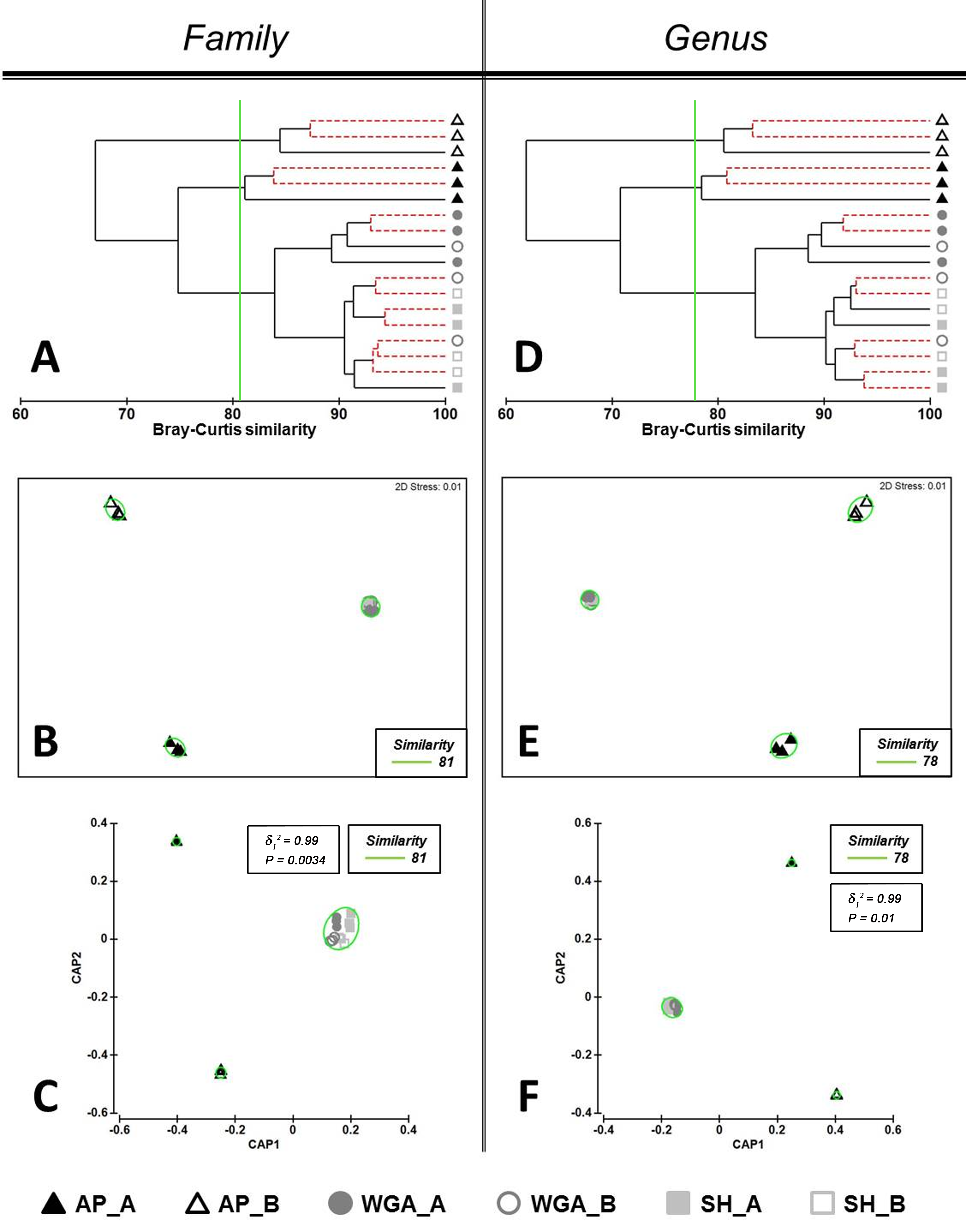

Supplement: Figure S3 — Comparison of the soil rRNA profiles generated on full datasets at the phylum (A, B, C) and class (D, E, F) taxonomic resolution levels. Bray-Curtis distance similarity matrix was calculated from the square-root transformed abundance of DNA fragments matching taxa in the M5RNA database (E-value <1×10^−5^). The Bray-Curtis matrix was used for generating CLUSTER dendrogram, NMDS and CAP ordination plots. CLUSTER analysis (A and D). Red dotted branches on the CLUSTER dendrogram indicate no significant difference between metagenomic profiles (supported by the SIMPROF analysis, p<0.05). NMDS unconstrained ordination (B and E). The NMDS plot displays distances between samples. Data points that are closer to each other represent samples with highly similar metagenomic profiles. CAP constrained ordination (C and F). CAP analysis tests for differences among the groups in multivariate space. The significance of group separation along the canonical axis is indicated by the value of the squared canonical correlation (δ1 2) and P-value (P<0.05). A contour line on the NMDS and CAP ordinations drawn round each of the cluster defines the superimposition of clusters from CLUSTER dendrogram at the selected level of similarity. (TIF) [file pone.0104996.s003.tif]

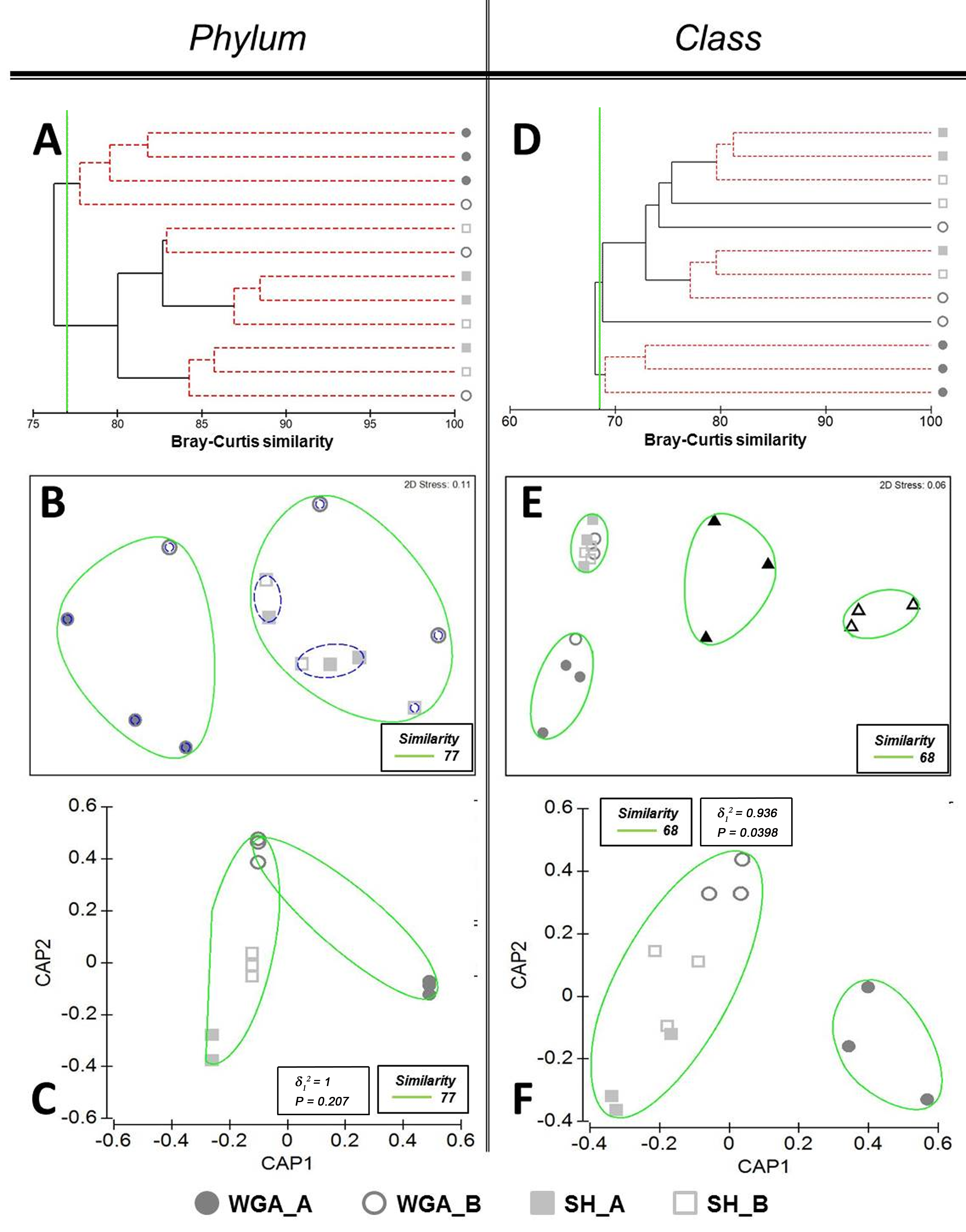

Supplement: Figure S4 — Comparison of the soil rRNA profiles generated on full datasets at the phylum (A, B, C) and class (D, E, F) taxonomic resolution levels. Bray-Curtis distance similarity matrix was calculated from the square-root transformed abundance of DNA fragments matching taxa in the M5RNA database (E-value <1×10^−5^). The Bray-Curtis matrix was used for generating CLUSTER dendrogram, NMDS and CAP ordination plots. CLUSTER analysis (A and D). Red dotted branches on the CLUSTER dendrogram indicate no significant difference between metagenomic profiles (supported by the SIMPROF analysis, p<0.05). NMDS unconstrained ordination (B and E). The NMDS plot displays distances between samples. Data points that are closer to each other represent samples with highly similar metagenomic profiles. CAP constrained ordination (C and F). CAP analysis tests for differences among the groups in multivariate space. The significance of group separation along the canonical axis is indicated by the value of the squared canonical correlation (δ1 2) and P-value (P<0.05). A contour line on the NMDS and CAP ordinations drawn round each of the cluster defines the superimposition of clusters from CLUSTER dendrogram at the selected level of similarity. (TIF) [file pone.0104996.s004.tif]

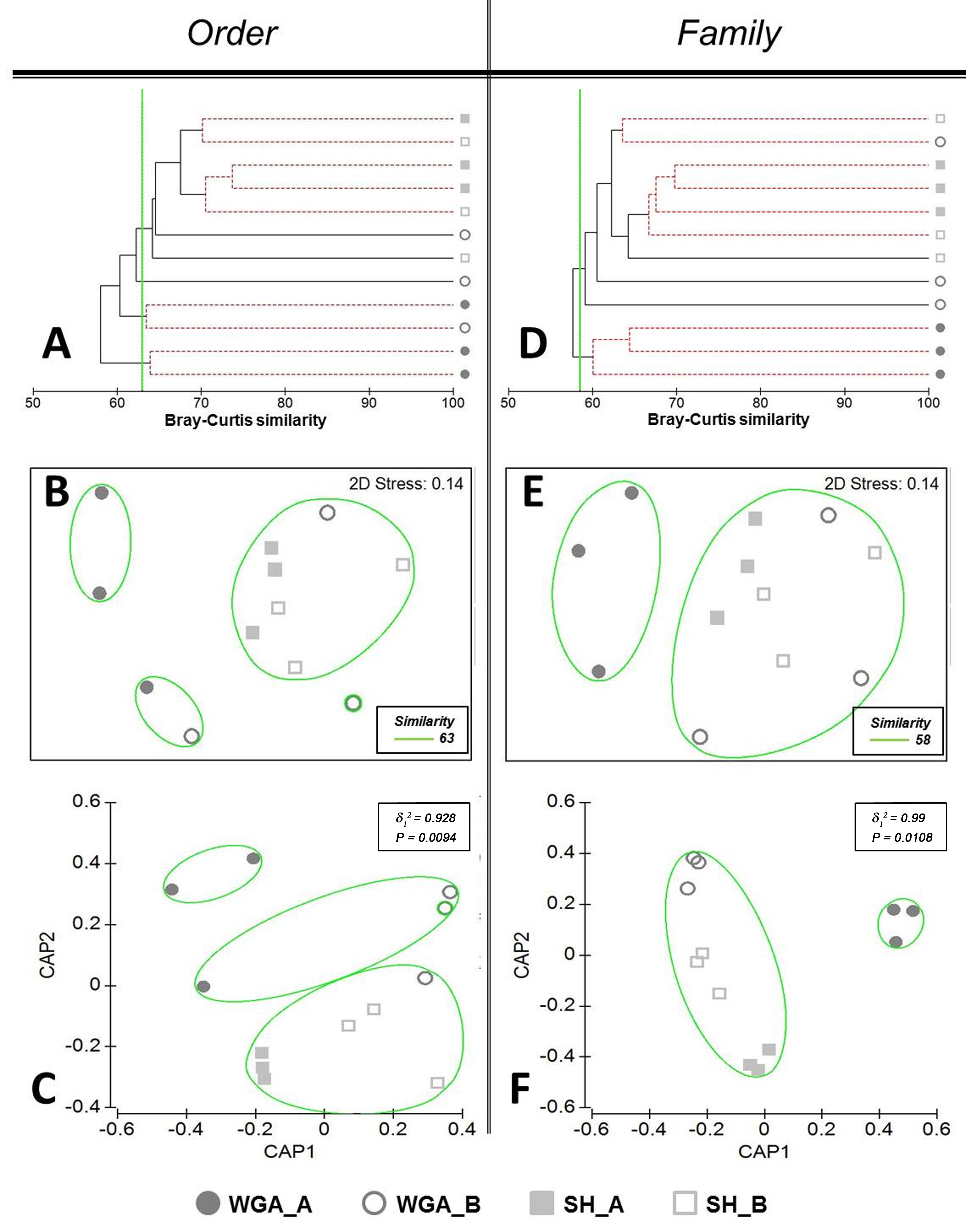

Supplement: Figure S5 — Comparison of the soil rRNA profiles generated on full datasets at the order (A, B, C) and family (D, E, F) taxonomic resolution levels. Bray-Curtis distance similarity matrix was calculated from the square-root transformed abundance of DNA fragments matching taxa in the M5RNA database (E-value <1×10^−5^). The Bray-Curtis matrix was used for generating CLUSTER dendrogram, NMDS and CAP ordination plots. CLUSTER analysis (A and D). Red dotted branches on the CLUSTER dendrogram indicate no significant difference between metagenomic profiles (supported by the SIMPROF analysis, p<0.05). NMDS unconstrained ordination (B and E). The NMDS plot displays distances between samples. Data points that are closer to each other represent samples with highly similar metagenomic profiles. CAP constrained ordination (C and F). CAP analysis tests for differences among the groups in multivariate space. The significance of group separation along the canonical axis is indicated by the value of the squared canonical correlation (δ1 2) and P-value (P<0.05). A contour line on the NMDS and CAP ordinations drawn round each of the cluster defines the superimposition of clusters from CLUSTER dendrogram at the selected level of similarity. (TIF) [file pone.0104996.s005.tif]

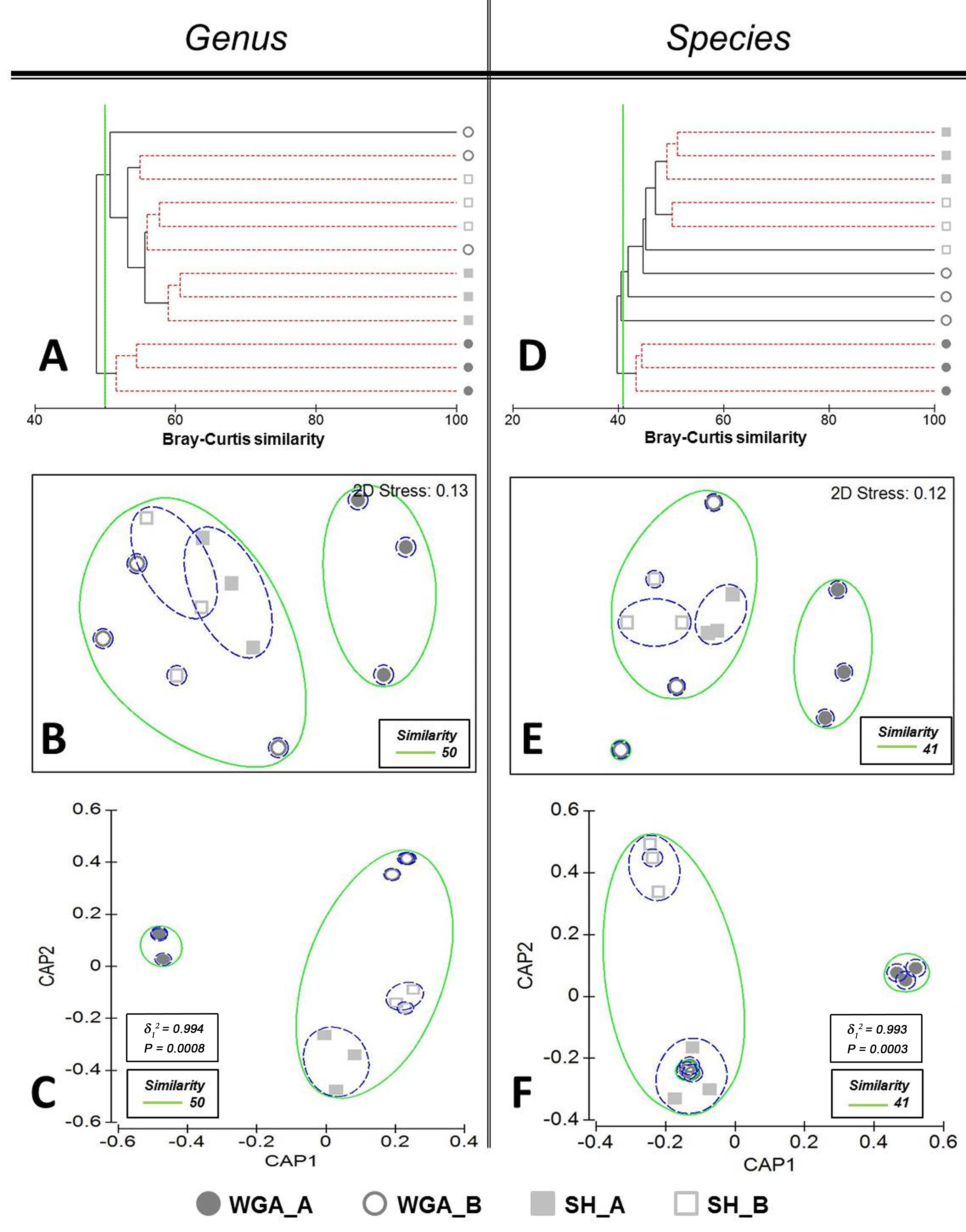

Supplement: Figure S6 — Comparison of the soil rRNA profiles generated on full datasets at the genus (A, B, C) and species (D, E, F) taxonomic resolution levels. Bray-Curtis distance similarity matrix was calculated from the square-root transformed abundance of DNA fragments matching taxa in the M5RNA database (E-value <1×10^−5^). The Bray-Curtis matrix was used for generating CLUSTER dendrogram, NMDS and CAP ordination plots. CLUSTER analysis (A and D). Red dotted branches on the CLUSTER dendrogram indicate no significant difference between metagenomic profiles (supported by the SIMPROF analysis, p<0.05). NMDS unconstrained ordination (B and E). The NMDS plot displays distances between samples. Data points that are closer to each other represent samples with highly similar metagenomic profiles. CAP constrained ordination (C and F). CAP analysis tests for differences among the groups in multivariate space. The significance of group separation along the canonical axis is indicated by the value of the squared canonical correlation (δ1 2) and P-value (P<0.05). A contour line on the NMDS and CAP ordinations drawn round each of the cluster defines the superimposition of clusters from CLUSTER dendrogram at the selected level of similarity. (TIF) [file pone.0104996.s006.tif]

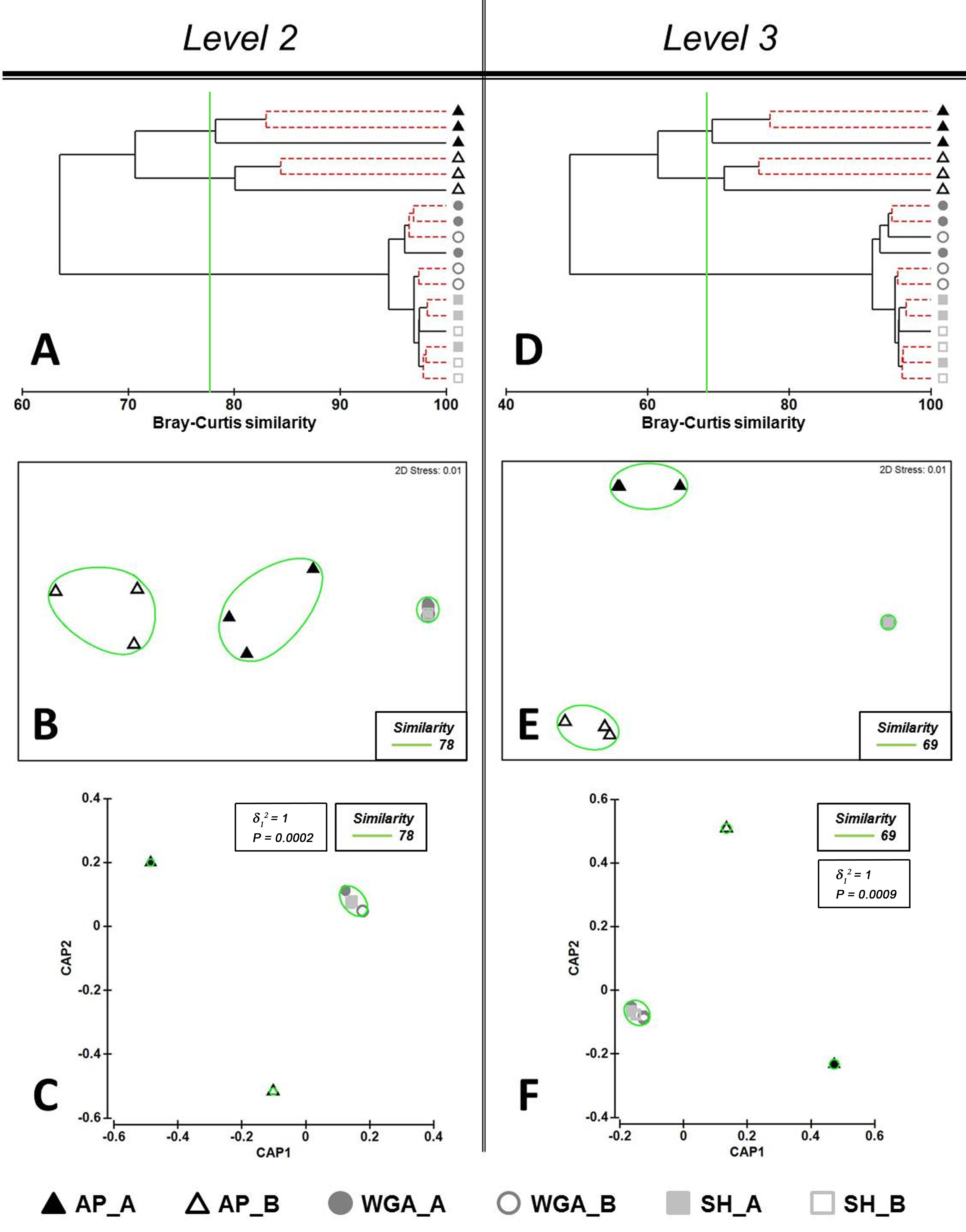

Supplement: Figure S7 — Comparison of the soil metabolic profiles generated on full datasets at the subsystems level 2 (A, B, C) and level 3 (D, E, F) metabolic resolution levels. Bray-Curtis distance similarity matrix was calculated from the square-root transformed abundance of DNA fragments matching taxa in the SEED database (E-value <1×10^−5^). The Bray-Curtis matrix was used for generating CLUSTER dendrogram, NMDS and CAP ordination plots. CLUSTER analysis (A and D). Red dotted branches on the CLUSTER dendrogram indicate no significant difference between metagenomic profiles (supported by the SIMPROF analysis, p<0.05). NMDS unconstrained ordination (B and E). The NMDS plot displays distances between samples. Data points that are closer to each other represent samples with highly similar metagenomic profiles. CAP constrained ordination (C and F). CAP analysis tests for differences among the groups in multivariate space. The significance of group separation along the canonical axis is indicated by the value of the squared canonical correlation (δ1 2) and P-value (P<0.05). A contour line on the NMDS and CAP ordinations drawn round each of the cluster defines the superimposition of clusters from CLUSTER dendrogram at the selected level of similarity. (TIF) [file pone.0104996.s007.tif]

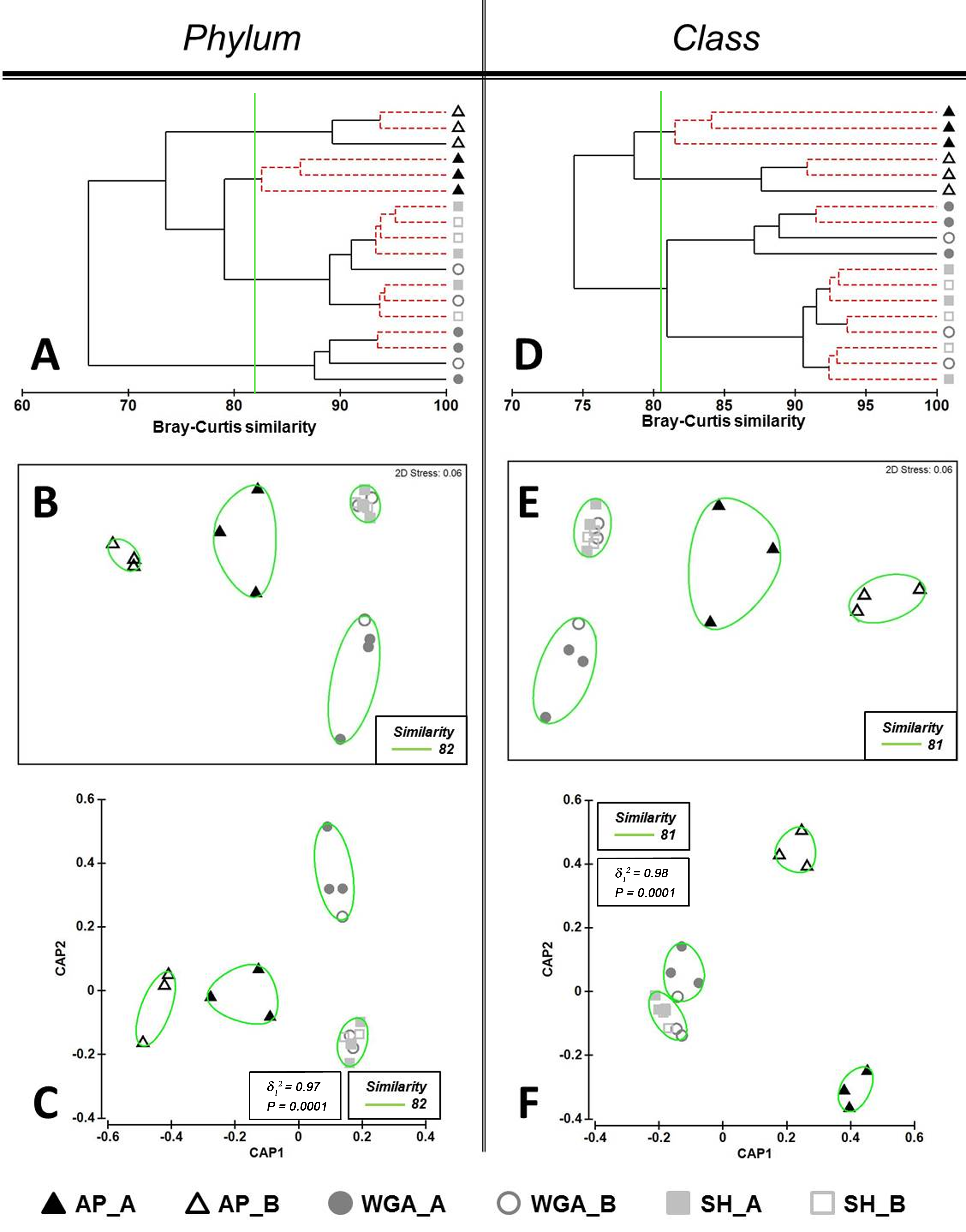

Supplement: Figure S8 — Comparison of the soil protein-derived taxonomic profiles generated on randomly sub-sampled datasets at the phylum (A, B, C) and class (D, E, F) metabolic resolution levels. Bray-Curtis distance similarity matrix was calculated from the square-root transformed abundance of DNA fragments matching taxa in the M5NR database (E-value <1×10^−5^). The Bray-Curtis matrix was used for generating CLUSTER dendrogram, NMDS and CAP ordination plots. CLUSTER analysis (A and D). Red dotted branches on the CLUSTER dendrogram indicate no significant difference between metagenomic profiles (supported by the SIMPROF analysis, p<0.05). NMDS unconstrained ordination (B and E). The NMDS plot displays distances between samples. Data points that are closer to each other represent samples with highly similar metagenomic profiles. CAP constrained ordination (C and F). CAP analysis tests for differences among the groups in multivariate space. The significance of group separation along the canonical axis is indicated by the value of the squared canonical correlation (δ1 2) and P-value (P<0.05). A contour line on the NMDS and CAP ordinations drawn round each of the cluster defines the superimposition of clusters from CLUSTER dendrogram at the selected level of similarity. (TIF) [file pone.0104996.s008.tif]

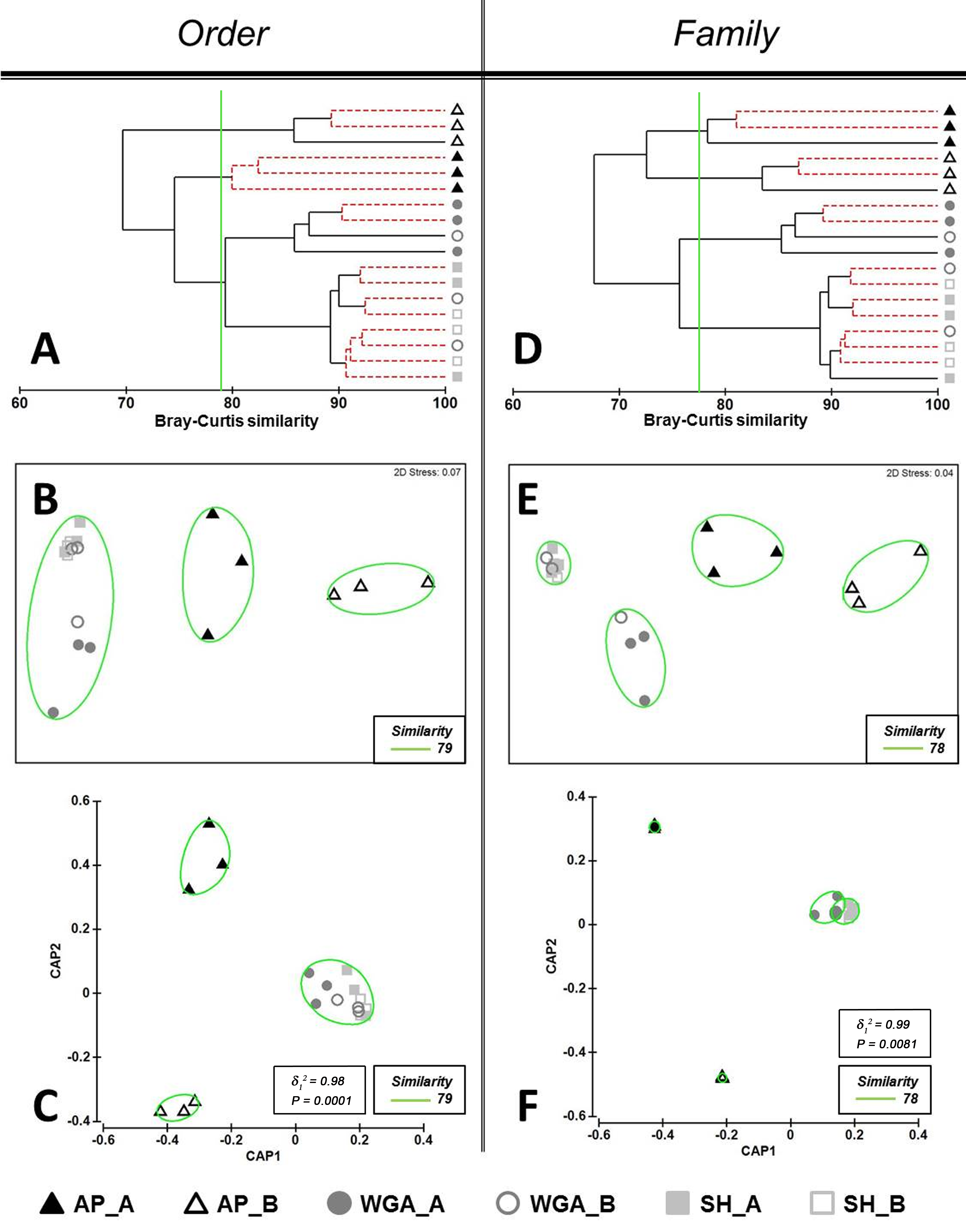

Supplement: Figure S9 — Comparison of the soil protein-derived taxonomic profiles generated on randomly sub-sampled datasets at the order (A, B, C) and family (D, E, F) taxonomic resolution levels. Bray-Curtis distance similarity matrix was calculated from the square-root transformed abundance of DNA fragments matching taxa in the SEED database (E-value <1×10^−5^). The Bray-Curtis matrix was used for generating CLUSTER dendrogram, NMDS and CAP ordination plots. CLUSTER analysis (A and D). Red dotted branches on the CLUSTER dendrogram indicate no significant difference between metagenomic profiles (supported by the SIMPROF analysis, p<0.05). NMDS unconstrained ordination (B and E). The NMDS plot displays distances between samples. Data points that are closer to each other represent samples with highly similar metagenomic profiles. CAP constrained ordination (C and F). CAP analysis tests for differences among the groups in multivariate space. The significance of group separation along the canonical axis is indicated by the value of the squared canonical correlation (δ1 2) and P-value (P<0.05). A contour line on the NMDS and CAP ordinations drawn round each of the cluster defines the superimposition of clusters from CLUSTER dendrogram at the selected level of similarity. (TIF) [file pone.0104996.s009.tif]

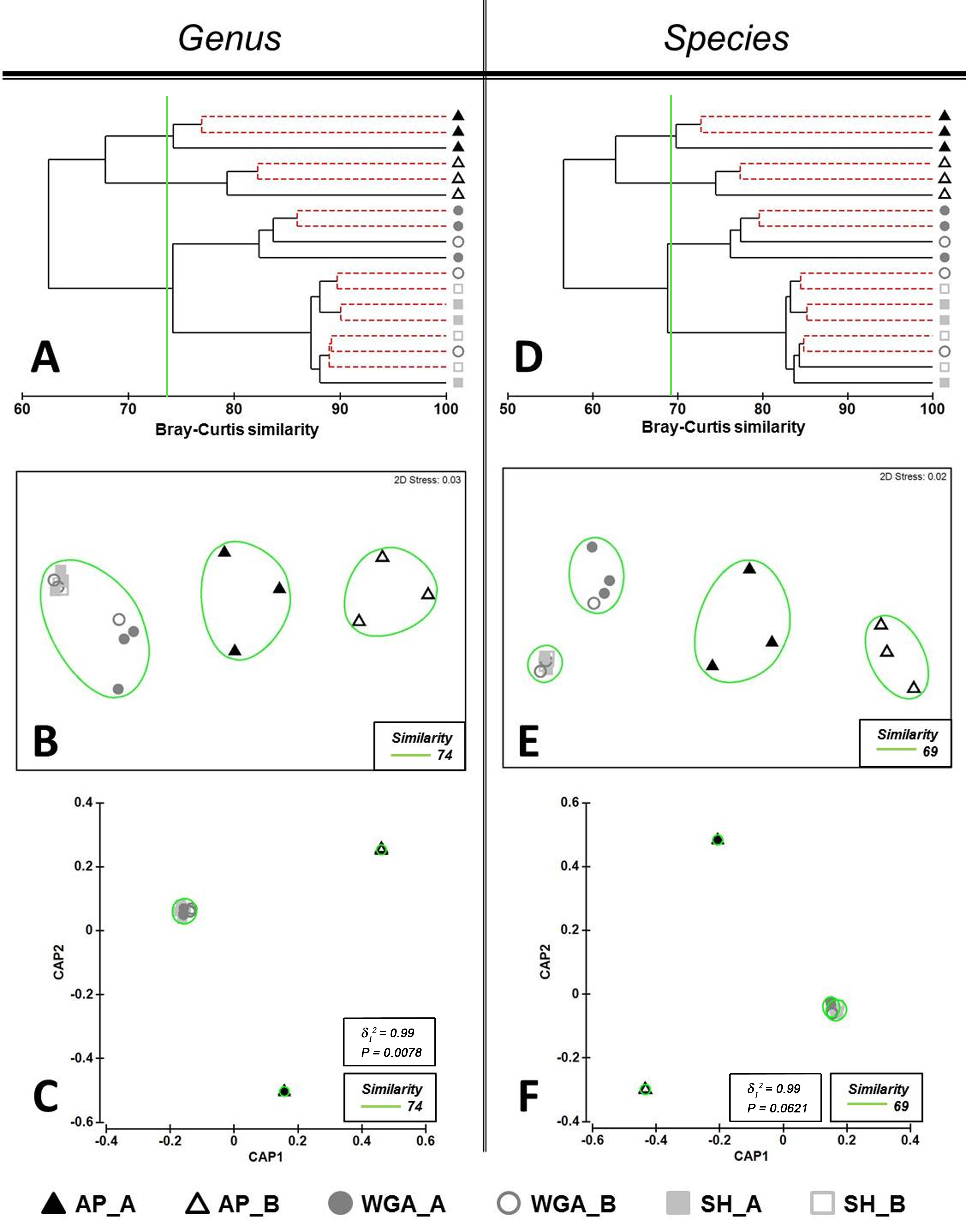

Supplement: Figure S10 — Comparison of the soil protein-derived taxonomic profiles generated on randomly sub-sampled datasets at the genus (A, B, C) and species (D, E, F) taxonomic resolution levels. Bray-Curtis distance similarity matrix was calculated from the square-root transformed abundance of DNA fragments matching taxa in the M5NR database (E-value <1×10^−5^). The Bray-Curtis matrix was used for generating CLUSTER dendrogram, NMDS and CAP ordination plots. CLUSTER analysis (A and D). Red dotted branches on the CLUSTER dendrogram indicate no significant difference between metagenomic profiles (supported by the SIMPROF analysis, p<0.05). NMDS unconstrained ordination (B and E). The NMDS plot displays distances between samples. Data points that are closer to each other represent samples with highly similar metagenomic profiles. CAP constrained ordination (C and F). CAP analysis tests for differences among the groups in multivariate space. The significance of group separation along the canonical axis is indicated by the value of the squared canonical correlation (δ1 2) and P-value (P<0.05). A contour line on the NMDS and CAP ordinations drawn round each of the cluster defines the superimposition of clusters from CLUSTER dendrogram at the selected level of similarity. (TIF) [file pone.0104996.s010.tif]

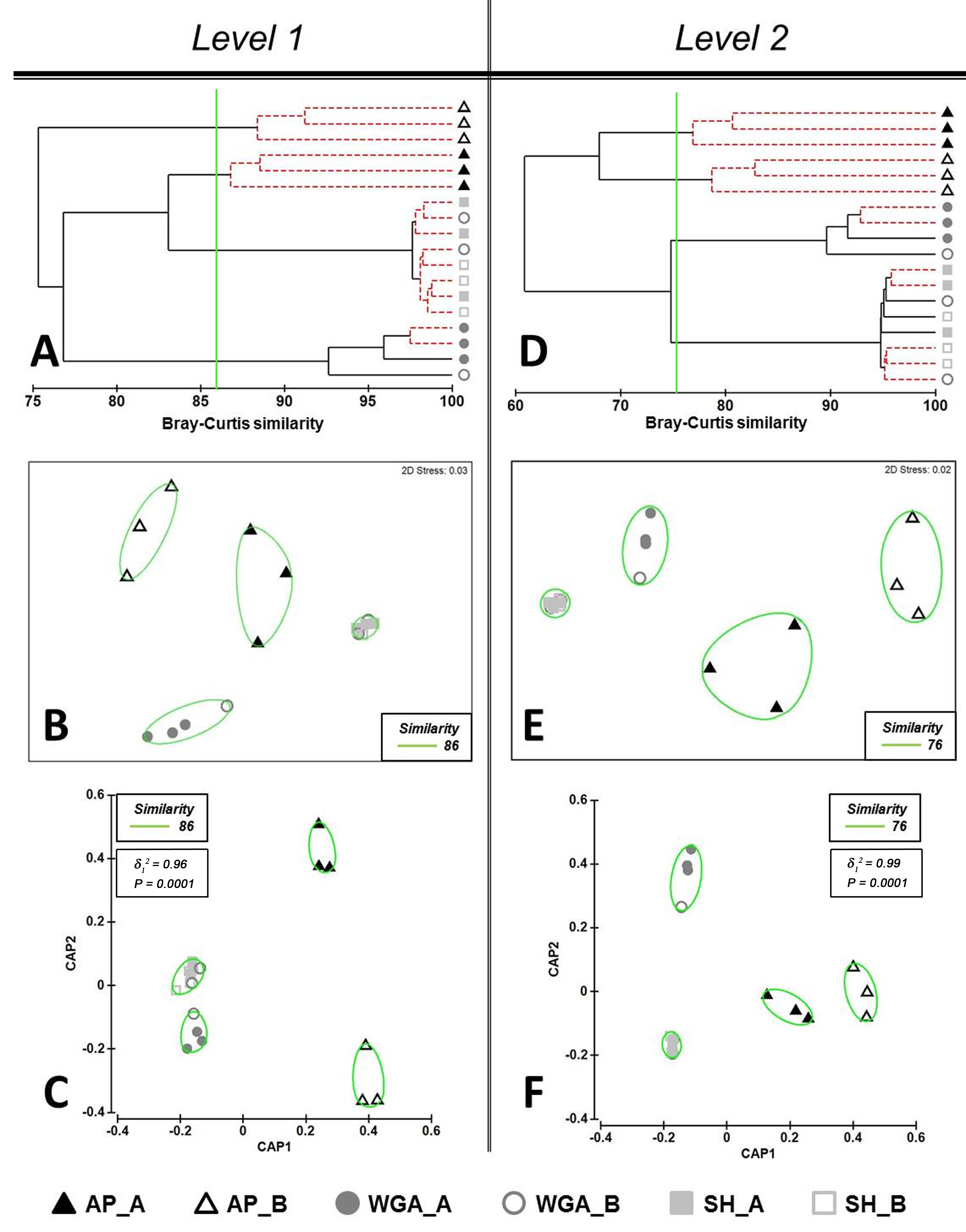

Supplement: Figure S11 — Comparison of the soil metabolic profiles generated on randomly sub-sampled datasets at the subsystems level 1 (A, B, C) and subsystems Level 2 (D, E, F) metabolic resolution levels. Bray-Curtis distance similarity matrix was calculated from the square-root transformed abundance of DNA fragments matching taxa in the SEED database (E-value <1×10^−5^). The Bray-Curtis matrix was used for generating CLUSTER dendrogram, NMDS and CAP ordination plots. CLUSTER analysis (A and D). Red dotted branches on the CLUSTER dendrogram indicate no significant difference between metagenomic profiles (supported by the SIMPROF analysis, p<0.05). NMDS unconstrained ordination (B and E). The NMDS plot displays distances between samples. Data points that are closer to each other represent samples with highly similar metagenomic profiles. CAP constrained ordination (C and F). CAP analysis tests for differences among the groups in multivariate space. The significance of group separation along the canonical axis is indicated by the value of the squared canonical correlation (δ1 2) and P-value (P<0.05)… A contour line on the NMDS and CAP ordinations drawn round each of the cluster defines the superimposition of clusters from CLUSTER dendrogram at the selected level of similarity. (TIF) [file pone.0104996.s011.tif]

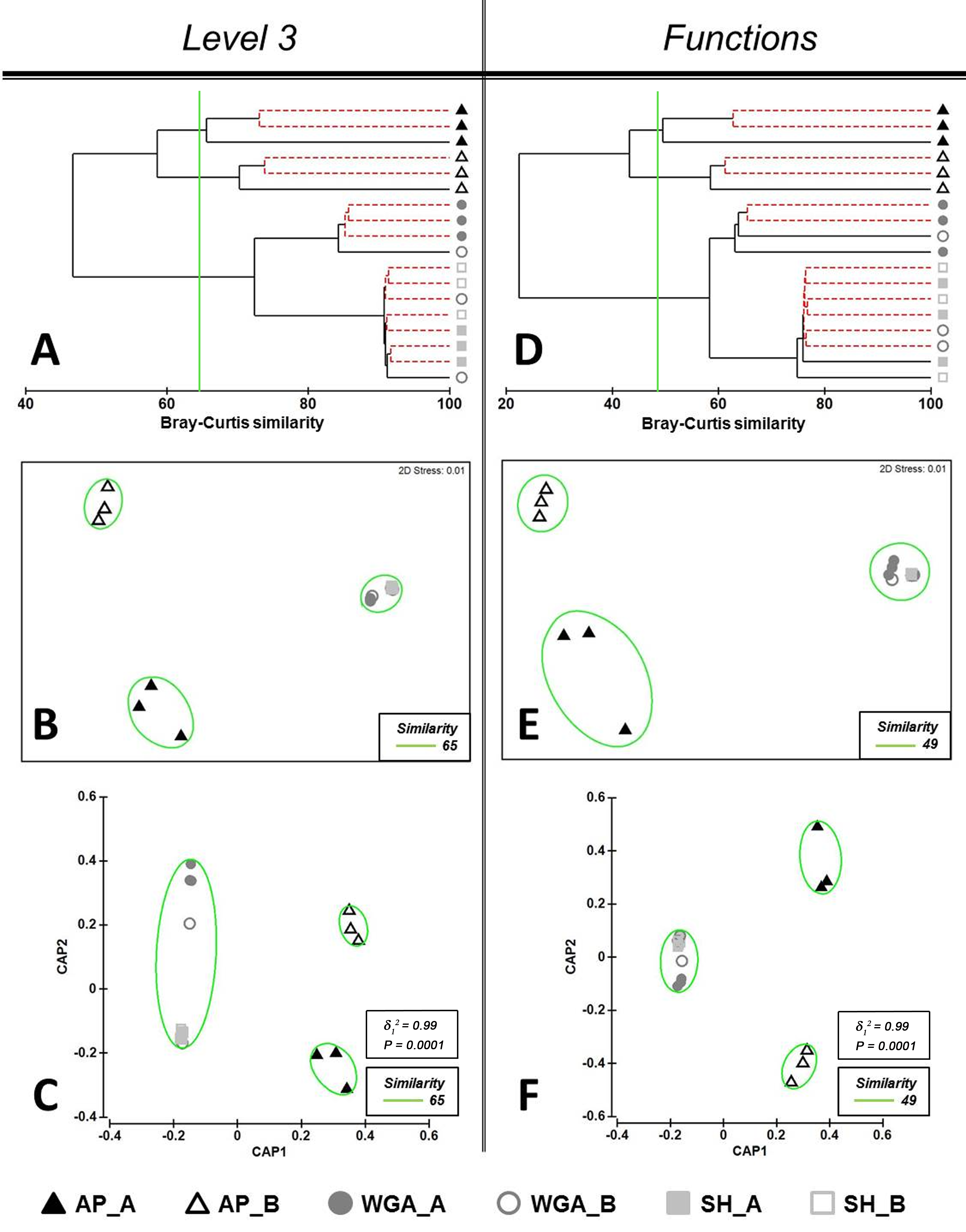

Supplement: Figure S12 — Comparison of the soil metabolic profiles generated on randomly sub-sampled datasets at the subsystems level 3 (A, B, C) and subsystems functions (D, E, F) metabolic resolution levels. Bray-Curtis distance similarity matrix was calculated from the square-root transformed abundance of DNA fragments matching taxa in the SEED database (E-value <1×10^−5^). The Bray-Curtis matrix was used for generating CLUSTER dendrogram, NMDS and CAP ordination plots. CLUSTER analysis (A and D). Red dotted branches on the CLUSTER dendrogram indicate no significant difference between metagenomic profiles (supported by the SIMPROF analysis, p<0.05). NMDS unconstrained ordination (B and E). The NMDS plot displays distances between samples. Data points that are closer to each other represent samples with highly similar metagenomic profiles. CAP constrained ordination (C and F). CAP analysis tests for differences among the groups in multivariate space. The significance of group separation along the canonical axis is indicated by the value of the squared canonical correlation (δ1 2) and P-value (P<0.05). A contour line on the NMDS and CAP ordinations drawn round each of the cluster defines the superimposition of clusters from CLUSTER dendrogram at the selected level of similarity. (TIF) [file pone.0104996.s012.tif]
