# Supplementary material for: Random Whole Metagenomic Sequencing for Forensic Discrimination of Soils
Source: PLoS One. 2014 Aug 11;9(8):e104996. doi: 10.1371/journal.pone.0104996 (PMC4128759; doi:10.1371/journal.pone.0104996)
Supplement: Table S1 — Summary of soil metagenomic samples. All metagenomes are publically available on the MG-RAST server (http://metagenomics.anl.gov/). (PDF) [file pone.0104996.s013.pdf]

Group	Full sequencing datasets		Randomly subsampled datasets	
	**Sample name**	**MG-RAST ID**	**Sample name**	**MG-RAST ID**
**SH_A**	**AKAn**	4533948.3	**An_AN**	4553173.3
	**AKAs**	4533949.3	**As_AN**	4553184.3
	**AKAw**	4533950.3	**Aw_AN**	4553185.3
**SH_B**	**AKBe**	4533951.3	**Be_AN**	4553186.3
	**AKBn**	4533952.3	**Bn_AN**	4553187.3
	**AKBs**	4533953.3	**Bs_AN**	4553188.3
**AP_A**	**APPCR_A4n**	4549132.3	**APPCR_An_AN**	4553174.3
	**APPCR_A4s**	4549136.3	**APPCR_As_AN**	4553178.3
	**APPCR_A4w**	4549137.3	**APPCR_Aw_AN**	4553179.3
**AP_B**	**APPCR_B4e**	4549141.3	**APPCR_Be_AN**	4553180.3
	**APPCR_B4n**	4549142.3	**APPCR_Bn_AN**	4553181.3
	**APPCR_B4s**	4549144.3	**APPCR_Bs_AN**	4553183.3
**WGA_A**	**AK_WGA_An**	4543715.3	**WGA_An_AN**	4553189.3
	**AK_WGA_As**	4543716.3	**WGA_As_AN**	4553190.3
	**AK_WGA_Aw**	4543717.3	**WGA_Aw_AN**	4553191.3
**WGA_B**	**AK_WGA_Be**	4543718.3	**WGA_Be_AN**	4553192.3
	**AK_WGA_Bn**	4543719.3	**WGA_Bn_AN**	4553193.3
	**AK_WGA_Bs**	4543720.3	**WGA_Bs_AN**	4553194.3
