# Supplementary material for: Random Whole Metagenomic Sequencing for Forensic Discrimination of Soils
Source: PLoS One. 2014 Aug 11;9(8):e104996. doi: 10.1371/journal.pone.0104996 (PMC4128759; doi:10.1371/journal.pone.0104996)
Supplement: Table S2 — Protein-derived taxonomic composition of the soil microbial communities. Relative abundances of major taxa (phylum level) derived from taxonomic assignment of protein gene fragments matched to M5NR database. (PDF) [file pone.0104996.s014.pdf]

M5NR		WGA						SH						AP					
domain	phylum	WGA_Aw	WGA_As	WGA_An	WGA_Bn	WGA_Bs	WGA_Be	SH_As	SH_An	SH_Aw	SH_Be	SH_Bn	SH_Bs	AP_An	AP_Aw	AP_As	AP_Bs	AP_Bn	AP_Be
Archaea	Thaumarchaeota	0.63	0.32	0.11	0.25	0.02	0.05	0.09	0.2	0.41	0.12	0.04	0.22	0	0	0	0.02	0	0
	Crenarchaeota	0.2	0.16	0.03	0.13	0.03	0.06	0.08	0.15	0.16	0.08	0.04	0.1	0.04	0.08	0.06	0.01	0.01	0.03
	Euryarchaeota	0.97	0.63	0.38	0.49	0.35	0.29	0.34	0.43	0.54	0.45	0.32	0.43	0.84	0.54	0.29	0.19	0.16	0.13
	Total	1.83	1.16	0.53	0.89	0.4	0.41	0.52	0.78	1.13	0.67	0.4	0.76	0.88	0.62	0.37	0.22	0.17	0.16
Bacteria	Verrucomicrobia	1.92	2.14	1.57	2.82	2.23	1.54	1.29	1.71	1.32	1.52	2.25	2.05	1.21	1.12	2.26	2.44	1.83	1.94
	Bacteroidetes	18.56	21.07	23.98	20.27	8.48	5.67	11.5	8.66	7.16	8.11	13.09	8.78	10.15	4.41	9.45	6.59	4.6	6.43
	Actinobacteria	14.35	12.31	11.2	9.42	28.1	41.56	26.02	30.27	34.04	33.8	19.77	25.86	11.93	24.45	12.33	7	9.84	11.73
	Chlorobi	0.68	0.55	0.48	0.48	0.18	0.22	0.27	0.35	0.3	0.3	0.31	0.36	0.34	0.1	0.16	1.16	1.74	1.38
	Nitrospirae	0.2	0.2	0.11	0.38	0.12	0.1	0.07	0.16	0.18	0.12	0.14	0.21	0.05	0.14	0.05	0.09	0.14	0.18
	Deinococcus-Thermus	0.59	0.37	0.45	0.46	0.38	0.46	0.38	0.47	0.54	0.4	0.44	0.41	0.56	0.48	0.25	0.55	0.29	0.47
	Gemmatimonadetes	0.17	0.11	0.12	0.16	0.22	0.21	0.14	0.21	0.32	0.26	0.27	0.34	0.64	0.28	0.59	2.9	5.25	4.22
	Acidobacteria	1.68	1.6	1.44	2.23	1.69	1.12	1.1	1.45	1.31	1.3	1.8	1.68	6.06	4.78	6.96	5.03	6.52	5.57
	Spirochaetes	0.22	0.25	0.24	0.27	0.11	0.12	0.14	0.12	0.19	0.16	0.18	0.23	0.02	0.08	0.04	0.06	0.02	0.03
	Firmicutes	3.84	3.6	2.61	3.01	2.02	2.1	2.33	2.87	2.98	2.5	2.5	2.82	2.81	2.18	1.34	0.66	0.75	1.06
	Chloroflexi	2.41	1.73	1.17	1.26	0.99	0.98	1.03	1.58	1.81	0.99	0.96	1.27	1.6	3.48	5.34	7.12	6.78	5.23
	Planctomycetes	2.46	3.01	1.26	3.42	2.8	2.31	1.5	2.84	2.64	2.87	3	3.21	0.88	4.57	5.75	1.84	1.87	2.05
	Proteobacteria	34.05	33.18	30.43	33.67	37.43	31.06	39.36	35.78	34.42	33.76	38.93	38.2	38.65	36.81	39.54	46.54	44.77	44.35
	Chlamydiae	0.11	0.07	0.05	0.09	0.07	0.07	0.07	0.08	0.05	0.08	0.08	0.07	0.04	0.02	0.02	0.01	0.02	0.02
	Cyanobacteria	2.48	2.25	1.4	1.71	1.16	1.06	1.03	1.34	1.61	1.16	1.25	1.29	1.26	1.47	1.12	1.02	0.77	0.88
	Total	84.89	83.66	77.41	80.76	86.73	89.26	87.06	88.81	89.87	88.12	85.95	87.78	77.03	86.08	85.74	83.44	85.54	85.95
Eukaryota	Ascomycota	2.45	4.13	10.99	6.76	3.74	1.89	3.25	1.12	0.61	2.05	3.37	1.99	6.05	1.08	2.03	2.17	0.55	1.49
	Streptophyta	0.29	0.36	0.24	0.23	0.12	0.14	0.18	0.2	0.17	0.16	0.19	0.18	0.3	0.47	1.43	2.6	1.71	1.92
	Chordata	0.48	0.44	0.49	0.25	0.2	0.25	0.25	0.32	0.15	0.3	0.19	0.2	0.1	0.1	0.03	0.04	0.01	0.02
	Basidiomycota	0.1	0.14	0.37	0.28	0.11	0.07	0.12	0.07	0.03	0.07	0.12	0.04	0.19	0.17	0.07	0.04	0.01	0.04
	Arthropoda	0.19	0.44	0.19	0.13	0.05	0.18	0.13	0.24	0.04	0.29	0.08	0.07	0.03	0.07	0.04	0.04	0	0.05
	Total	4.07	6.13	12.83	8.18	4.53	2.85	4.24	2.25	1.28	3.24	4.31	2.83	6.85	2.14	3.69	5.02	2.35	3.61
