# Supplementary material for: Random Whole Metagenomic Sequencing for Forensic Discrimination of Soils
Source: PLoS One. 2014 Aug 11;9(8):e104996. doi: 10.1371/journal.pone.0104996 (PMC4128759; doi:10.1371/journal.pone.0104996)
Supplement: Table S4 — Metabolic composition of the soil microbial communities. Relative abundances of major metabolic features (level 1) derived from annotation of protein gene fragments to SEED Subsystems database. (PDF) [file pone.0104996.s016.pdf]

| SEED Subsystems | WGA | | | | | | SH | | | | | | AP | | | | | |
|---|---|---|---|---|---|---|---|---|---|---|---|---|---|---|---|---|---|---|
| Level 1 | WGA_Aw | WGA_As | WGA_An | WGA_Bn | WGA_Bs | WGA_Be | SH_As | SH_An | SH_Aw | SH_Be | SH_Bn | SH_Bs | AP_An | AP_Aw | AP_As | AP_Bs | AP_Bn | AP_Be |
| Clustering-based subsystems | 14.3 | 14.4 | 15.5 | 14.8 | 15.0 | 14.8 | 14.7 | 15.1 | 15.6 | 15.0 | 15.1 | 15.0 | 11.3 | 9.6 | 13.7 | 17.9 | 17.1 | 16.4 |
| Carbohydrates | 12.5 | 12.1 | 12.1 | 12.5 | 12.1 | 13.5 | 11.8 | 12.4 | 12.9 | 13.2 | 11.2 | 12.3 | 18.5 | 19.9 | 18.4 | 14.9 | 13.3 | 16.6 |
| Amino Acids and Derivatives | 8.4 | 8.0 | 7.9 | 8.3 | 8.9 | 9.0 | 8.7 | 8.5 | 8.2 | 8.4 | 8.4 | 8.4 | 10.5 | 7.1 | 6.0 | 8.1 | 5.3 | 6.6 |
| Protein Metabolism | 7.4 | 8.1 | 8.2 | 7.8 | 8.0 | 7.6 | 7.5 | 7.7 | 7.8 | 7.6 | 7.5 | 8.2 | 9.1 | 7.1 | 17.3 | 17.9 | 16.4 | 16.7 |
| Miscellaneous | 7.0 | 7.2 | 7.7 | 7.2 | 7.0 | 6.9 | 8.3 | 8.2 | 7.8 | 8.3 | 7.9 | 8.0 | 7.0 | 9.5 | 7.1 | 4.7 | 3.8 | 4.7 |
| Cofactors, Vitamins, Prosthetic Groups, Pigments | 5.4 | 6.2 | 6.2 | 6.1 | 6.2 | 6.6 | 5.9 | 6.4 | 6.1 | 6.4 | 6.3 | 6.3 | 7.6 | 5.6 | 12.3 | 18.8 | 20.6 | 15.5 |
| DNA Metabolism | 4.7 | 4.5 | 4.2 | 4.2 | 4.0 | 3.6 | 4.1 | 3.8 | 3.9 | 3.7 | 4.2 | 3.9 | 8.5 | 3.9 | 1.2 | 1.6 | 1.4 | 1.4 |
| RNA Metabolism | 3.7 | 3.7 | 3.8 | 3.9 | 3.6 | 3.2 | 3.2 | 3.3 | 3.2 | 3.4 | 3.8 | 3.4 | 2.7 | 2.6 | 1.2 | 1.1 | 0.4 | 0.8 |
| Respiration | 3.5 | 3.1 | 2.2 | 2.5 | 1.5 | 1.3 | 1.7 | 1.6 | 1.4 | 1.5 | 1.6 | 1.7 | 0.6 | 0.6 | 0.4 | 0.2 | 0.2 | 0.5 |
| Membrane Transport | 3.4 | 3.3 | 3.7 | 3.5 | 3.4 | 3.1 | 3.7 | 3.8 | 3.5 | 3.5 | 3.8 | 3.3 | 5.4 | 3.4 | 5.7 | 4.5 | 2.8 | 3.8 |
| Cell Wall and Capsule | 3.4 | 4.0 | 3.6 | 3.2 | 3.2 | 3.2 | 3.7 | 3.5 | 3.2 | 3.3 | 3.5 | 3.1 | 1.3 | 4.0 | 2.0 | 0.9 | 0.9 | 1.1 |
| Fatty Acids, Lipids, and Isoprenoids | 3.1 | 3.1 | 3.3 | 3.5 | 3.4 | 3.9 | 3.5 | 3.8 | 3.8 | 3.4 | 3.3 | 3.6 | 4.6 | 8.4 | 3.3 | 0.9 | 0.9 | 0.9 |
| Stress Response | 2.9 | 3.1 | 2.8 | 3.1 | 2.8 | 2.5 | 2.6 | 2.4 | 2.5 | 2.5 | 3.1 | 2.9 | 0.5 | 2.5 | 0.8 | 0.3 | 0.3 | 0.6 |
| Nucleosides and Nucleotides | 2.8 | 2.7 | 2.8 | 2.8 | 3.4 | 3.5 | 3.3 | 3.2 | 3.4 | 3.4 | 3.4 | 3.2 | 1.3 | 2.2 | 1.1 | 0.4 | 0.3 | 0.4 |
| Virulence, Disease and Defense | 2.7 | 2.5 | 2.5 | 2.6 | 3.0 | 2.7 | 2.9 | 2.7 | 2.7 | 2.9 | 2.7 | 2.9 | 2.0 | 1.4 | 1.2 | 1.1 | 0.9 | 0.7 |
| Metabolism of Aromatic Compounds | 2.7 | 2.8 | 2.8 | 2.7 | 2.9 | 3.0 | 2.9 | 2.7 | 2.6 | 2.7 | 2.6 | 2.7 | 3.2 | 4.4 | 3.2 | 3.6 | 8.6 | 7.0 |
| Other | 12.3 | 11.4 | 10.9 | 11.1 | 11.7 | 11.6 | 11.5 | 11.0 | 11.3 | 10.9 | 11.5 | 11.1 | 5.8 | 7.7 | 5.1 | 3.2 | 6.8 | 6.4 |
