# Supplementary material for: Random Whole Metagenomic Sequencing for Forensic Discrimination of Soils
Source: PLoS One. 2014 Aug 11;9(8):e104996. doi: 10.1371/journal.pone.0104996 (PMC4128759; doi:10.1371/journal.pone.0104996)
Supplement: Table S5 — Results of CAP model cross-validation of soil protein-derived taxonomic profiles discrimination generated from sub-sampled sequencing datasets. (PDF) [file pone.0104996.s017.pdf]

Original Group	AP_A	AP_B	WGA_A	WGA_B	SH_A	SH_B
Taxonomy level	***phylum*** (m = 2, δ_1^2 = 0.97, P = 0.0001)					
% correct	100	100	100	0	33	33
correct/total	3/3	3/3	3/3	0/3	1/3	1/3
Misclassified to group	n/a	n/a	n/a	SH_A<br>SH_B<br>WGA_A	SH_B	SH_A
Taxonomy level	***class*** (m = 5, δ_1^2 = 0.98, P = 0.0001)					
% correct	100	100	100	0	67	67
correct/total	3/3	3/3	3/3	0/3	2/3	2/3
Misclassified to group	n/a	n/a	n/a	SH_B<br>WGA_A	SH_B	WGA_B
Taxonomy level	***order*** (m = 3, δ_1^2 = 0.98, P = 0.0001)					
% correct	100	100	100	0	33	67
correct/total	3/3	3/3	3/3	0/3	1/3	2/3
Misclassified to group	n/a	n/a	n/a	SH_B<br>WGA_A	SH_B	SH_A
Taxonomy level	***family*** (m = 9, δ_1^2 = 0.99, P = 0.0081)					
% correct	100	100	100	0	67	67
correct/total	3/3	3/3	3/3	0/3	2/3	2/3
Misclassified to group	n/a	n/a	n/a	SH_B<br>WGA_A<br>SH_A	SH_B	WGA_B
Taxonomy level	***genus*** (m = 10, δ_1^2 = 0.99, P = 0.0078)					
% correct	100	100	100	0	67	67
correct/total	3/3	3/3	3/3	0/3	2/3	2/3
Misclassified to group	n/a	n/a	n/a	SH_B<br>WGA_A<br>SH_A	SH_B	SH_A
Taxonomy level	***species*** (m = 10, δ_1^2 = 0.99, P = 0.0621)					
% correct	100	100	100	0	67	67
correct/total	3/3	3/3	3/3	0/3	2/3	2/3
Misclassified to group	n/a	n/a	n/a	SH_B<br>WGA_A<br>SH_A	SH_B	SH_A
