# Supplementary material for: Random Whole Metagenomic Sequencing for Forensic Discrimination of Soils
Source: PLoS One. 2014 Aug 11;9(8):e104996. doi: 10.1371/journal.pone.0104996 (PMC4128759; doi:10.1371/journal.pone.0104996)
Supplement: Table S6 — Results of CAP model cross-validation of soil metabolic profiles discrimination generated from sub-sampled sequencing datasets. (PDF) [file pone.0104996.s018.pdf]

Original Group		AP_A	AP_B	WGA_A	WGA_B	SH_A	SH_B
Metabolic level	***level 1*** (m = 5, δ_1^2= 0.96, P = 0.0001)						
% correct		100	100	100	0	67	67
correct/total		3/3	3/3	3/3	0/3	2/3	2/3
Misclassified to group		n/a	n/a	n/a	SH_A<br>SH_B<br>WGA_A	SH_B	SH_A
Metabolic level	***level 2*** (m = 2, δ_1^2 = 0.99, P = 0.0001)						
% correct		100	100	100	0	67	100
correct/total		3/3	3/3	3/3	0/3	2/3	3/3
Misclassified to group		n/a	n/a	n/a	SH_B<br>WGA_A<br>SH_A	SH_B	n/a
Metabolic level	***level 3*** (m = 2, δ_1^2 = 0.99, P = 0.0001)						
% correct		100	100	100	0	67	100
correct/total		3/3	3/3	3/3	0/3	2/3	3/3
Misclassified to group		n/a	n/a	n/a	SH_B<br>WGA_A<br>SH_A	SH_B	n/a
Metabolic level	***functions*** (m = 3, δ_1^2 = 0.99, P = 0.0001)						
% correct		100	100	100	0	33	100
correct/total		3/3	3/3	3/3	0/3	1/3	3/3
Misclassified to group		n/a	n/a	n/a	SH_B<br>WGA_A<br>SH_B	SH_B	n/a
