# Supplementary material for: Random Whole Metagenomic Sequencing for Forensic Discrimination of Soils
Source: PLoS One. 2014 Aug 11;9(8):e104996. doi: 10.1371/journal.pone.0104996 (PMC4128759; doi:10.1371/journal.pone.0104996)
Supplement: Table S7 — Results of CAP model cross-validation of soil rRNA taxonomic profiles discrimination generated from full sequencing datasets. (PDF) [file pone.0104996.s019.pdf]

Original Group		WGA_A	WGA_B	SH_A	SH_B
Taxonomy level	***phylum*** (m = 9, δ_1^2 = 1, P = 0.207)				
% correct		100	33	33	0
correct/total		3/3	1/3	1/3	0/3
Misclassified to group		n/a	SH_B	SH_B	SH_A<br>WGA_B
Taxonomy level	***class*** (m = 5, δ_1^2 = 0.936, P = 0.039)				
% correct		33	0	33	100
correct/total		1/3	0/3	1/3	3/3
Misclassified to group		SH_B	SH_A<br>SH_B	SH_B	n/a
Taxonomy level	***order*** (m = 4, δ_1^2 = 0.928, P = 0.009)				
% correct		66	0	66	100
correct/total		2/3	0/3	2/3	3/3
Misclassified to group		SH_B	SH_A<br>SH_B	SH_B	n/a
Taxonomy level	***family*** (m = 6, δ_1^2 = 0.99, P = 0.0108)				
% correct		66	0	66	66
correct/total		2/3	0/3	2/3	2/3
Misclassified to group		SH_B	SH_B	SH_B	WGA_B
Taxonomy level	***genus*** (m = 8, δ_1^2 = 099, P = 0.0008)				
% correct		100	0	100	33
correct/total		3/3	0/3	3/3	1/3
Misclassified to group		n/a	SH_A<br>SH_B	n/a	SH_A<br>WGA_B
Taxonomy level	***species*** (m = 8, δ_1^2 = 0.99, P = 0.003)				
% correct		100	0	66	33
correct/total		3/3	0/3	2/3	1/3
Misclassified to group		n/a	SH_B	SH_B	SH_A<br>WGA_B
